# Long-term plastic mulching exacerbates the co-limitation of carbon and phosphorus in farmland by altering physicochemical properties and microbial interactions

**DOI:** 10.3389/fmicb.2025.1694370

**Published:** 2026-01-14

**Authors:** Tong Xu, Shuang Zheng, Xinqu Duo, Zhonghua Hou, Jinggui Wu

**Affiliations:** 1 College of Resource and Environmental Science, Jilin Agricultural University, Changchun, China; 2 Institute of Agricultural Resources and Environment, Jilin Academy of Agricultural Sciences, Changchun, China

**Keywords:** co-occurrence network, enzyme stoichiometry, microbial community, microplastic, nutrient limitation

## Abstract

Prolonged plastic film mulching causes plastic residue accumulation and microplastic (MP) formation, compromising soil structure and causing contamination. This study examined mulching duration effects (0, 5, 10, 15 years) on soil MPs, physicochemical properties, microbial communities, and nutrient limitations at 0–20 cm and 20–40 cm depths in maize soils of western Jilin, China. Mulching duration significantly increased MP abundance. Film-like MPs dominated, progressively fragmenting into smaller sizes over time. Long-term mulching enhanced soil moisture and EC (Electrical Conductivity) but decreased SOC (Soil Organic Carbon) and TN (Total Nitrogen), while increasing TP (Total Phosphorus) and AP (Available phosphorus). Microbial responses diverged: bacterial diversity and network complexity rose with enhanced cooperation, whereas fungal networks showed intensified competition. Extracellular enzyme stoichiometry indicated aggravated microbial co-limitation by C (Carbon) and P (Phosphorus), driven by MP-induced SOC depletion and altered P dynamics. SEM (Structural Equation Modeling) revealed that plastic mulching directly altered soil physicochemical properties through MPs accumulation, while indirectly regulating microbial community composition, ultimately exacerbating C-P co-limitation in microbial metabolism. The study highlights soil health risks from long-term mulching and highlights the necessity to seek alternatives such as biodegradable films to mitigate soil health risks associated with long-term plastic mulching.

## 1 Introduction

Mulching film emerged as a pivotal technology in 20th–century agricultural production, significantly advancing the development of human agricultural practices (Steinmetz et al., 2016). Its benefits include enhancing soil temperature and moisture retention, improving the efficiency of water and nutrient utilization, and expanding the cultivable area for crops (Xue et al., 2023). However, most of the existing thin mulch films tend to break during the recycling process, resulting in a low recovery rate. Consequently, residual mulch films accumulate in the soil. Plastics that enter the soil gradually degrade into plastic fragments or microbeads through physical fragmentation, chemical decomposition, or biodegradation (Surendran et al., 2023). MPs (Microplastics), defined as fragments or particles measuring less than 5 mm in diameter, possess distinct characteristics attributable to their small size and extensive specific surface area (Thompson et al., 2004). This unique structure significantly enhances their ability to adsorb a diverse range of pollutants from their surrounding environments. Consequently, MPs serve a crucial role as vectors of environmental pollution, underscoring their importance in discussions regarding ecological impacts and contamination (Song et al., 2024; Zhou J. et al., 2024).

MPs in agricultural ecosystems can greatly interfere with crucial ecological functions (Wang S. et al., 2022; Cao et al., 2023). The migration process of MPs within the soil is intricate, influenced by various factors such as soil characteristics, farming practices, plant growth processes, soil animal activities (including predation and reproduction), and the complexity of food webs (Zhang H. et al., 2022; Chen et al., 2024). MPs particles, ranging in size from 0.1 to 6 mm, can migrate through soil pores via leaching (Gao et al., 2019). Earthworms serve as a significant medium for the migration of MPs within the soil. These organisms may adhere to and ingest MPs, thereby facilitating their vertical migration (Qi et al., 2018). MPs contain harmful substances and can adsorb additional pollutants, leading to composite pollution that alters the physicochemical properties of soil, thereby harming the soil ecosystem. (An et al., 2024; Tang et al., 2024). Moreover, MPs negatively impact the soil nitrogen cycle, organic carbon dynamics, soil microbial activity, and nutrient transfer (Ma et al., 2023; Sun et al., 2023; Zhang et al., 2024). The addition of MPs alters the diversity and structure of microbial communities. Furthermore, plastics provide a new ecological niche for microorganisms, known as the plastisphere. Compared to natural habitats, the plastisphere aggregates and forms a unique microbial community.

Soil extracellular enzymes, primarily derived from animals, plants, and microorganisms, play an indispensable role in material cycling and energy flow. These enzymes can be categorized into hydrolases and oxidases. For instance, β-1, 4-glucosidase (BG) and β-D-cellobiohydrolase (CBH) are primarily involved in carbon acquisition, whereas β-1, 4-N-acetylglucosaminidase (NAG) and leucine aminopeptidase (LAP) are mainly utilized for nitrogen acquisition. Additionally, acid (or alkaline) phosphatase (ALP) is predominantly responsible for phosphorus acquisition. These enzymes catalyze the production of bioavailable terminal monomers, effectively reflecting the energy and nutrient metabolism levels of soil microorganisms (Li J. et al., 2024). Soil enzymatic stoichiometry provides a quantitative assessment of soil quality conditions, as well as the energy balance and dynamic processes within ecosystems, including nutrient decomposition and replenishment rates. This methodology serves as a crucial tool for investigating the interrelationships among carbon (C), nitrogen (N), phosphorus (P), and other elements in ecosystem dynamics. The presence of elements like C, N, and P often restricts the proliferation and functioning of microbial communities within soil in natural ecosystems, a situation known as microbial resource limitation (Yu et al., 2024). As research in ecological stoichiometry advances, the stoichiometric characteristics of soil extracellular enzymes and the activity ratios of these enzymes, specifically (CBH + BG): (NAG + LAP): AP, have emerged as significant areas of investigation. These metrics effectively reflect the robustness of microbial metabolism and the capacity of microbes to acquire energy and nutrients (Wang et al., 2021). The vector length (VL) and vector angle (VA) of enzyme measurements are utilized to indicate the relative energy and nutrient limitations encountered by microorganisms. A larger VL signifies a greater relative C limitation, while VA values less than 45° and greater than 45° represent relative degrees of N and P restrictions, respectively (Cui et al., 2022).

Soil microorganisms and enzymes are essential for energy flow and material cycling within the soil. They serve as key indicators for assessing soil fertility and are integral components of the soil ecosystem (Li Y. et al., 2024). Increasing attention is being drawn to the impact of MPs on the structural characteristics of soil microbial communities. Studies have indicated that fragments of PE (Polyethylene) can change microbial communities in soil and reduce microbial diversity. In contrast, PLA (polylactic acid) is known to improve bacterial diversity, which in turn affects the physicochemical properties of the soil (Lu et al., 2023). Additionally, the presence of PVC (Polyvinyl chloride) alters microbial community structure and metabolic states, shifting the community from Gram-positive to Gram-negative bacteria, which indicates that PVC addition promotes the soil carbon cycle (Shah et al., 2023). The presence of PP (Polypropylene) or PVC greatly diminishes the variety and quantity of bacterial communities found in acidic agricultural soils (Fei et al., 2020). During the early phases of soil cultivation experiments, a significant rise in the variety of bacterial communities was noted in soils influenced by PE (Ren et al., 2020).

To enhance maize production, plastic film mulching technology is extensively employed in the area (Bai and Gao, 2021). Nevertheless, after prolonged use and extensive application of mulch film, the inherent properties of the mulch result in a low recovery rate, leading to increasingly significant issues related to residual film pollution. This situation inevitably contributes to MP contamination, which, as an exogenous pollutant, may adversely affect soil properties and nutrient levels, thereby limiting microbial activity and metabolism. This research collected soil samples from maize farmland in western Jilin Province, characterized by varying mulching durations. The primary objectives were: (i) to identify the forms and abundance of MPs across different mulching years; (ii) to investigate the effects of MPs on the physicochemical properties of the soil; and (iii) to explore the influence of MPs on microbial nutrient limitations and community structure. This study aims to explore the impact of long-term film-mulching agricultural practices on farmland soil quality and to provide a scientific basis for sustainable agricultural production.

## 2 Materials and methods

### 2.1 Study site and sampling

The Qian’an Experimental Station, which is a component of the Jilin Academy of Agricultural Sciences, was the site for this research. Situated in Fuzi Village within Zanzi Town of Qian’an County, Songyuan, Jilin Province (N: 45° 01‵, E: 124° 02′), this locale is recognized as a typical semi-arid zone of Jilin Province and displays a mid-temperate continental monsoon climate. Significant climatic elements involve an average annual temperature of 5.6°C, a total of 2866.6 h of sunshine, and a yearly accumulated temperature of 2884.5°C. There are about 146 frost-free days on average each year, with annual precipitation reaching around 425.8 mm. The prevailing soil type in this area is identified as chernozem.

In the experimental station, maize fields with different mulching durations-5 years (Y5), 10 years (Y10), and 15 years (Y15)- were selected as treatment groups, while adjacent non-mulched fields (CK) served as controls. A fully randomized block design consisting of three replicates was utilized Details of the experimental treatments are provided in Supplementary Table 1. All fields followed identical agronomic practices: mulched plots were covered with transparent low-density polyethylene (LDPE) film (thickness: 0.008 mm, width: 150 cm), applied annually in mid-April. Weeds, diseases, and pests were effectively managed throughout the complete growing season. At the time of sowing, fertilizers were applied, including 63 kg ha^–1^ of N, 92 kg ha^–1^ of P (P_2_O_5_), and 80 kg ha^–1^ of K (K_2_O). An additional nitrogen application of 180 kg ha^–1^ took place during the 6-leaf stage. Drip irrigation was utilized, featuring four applications throughout the growing season, with each application delivering 40 mm of water. Maize was seeded at a plant density of 65,000 plants ha^–1^.

Soil samples were collected in August 2023, right after the maize harvest was completed. In order to ensure a representative analysis, five sampling points were chosen at random within each distinct plot. These points were arranged in an “S”-shaped pattern to minimize bias in sampling. The samples were taken from two specific depths: the top layer of soil ranging from 0 to 20 centimeters, known as the plow layer, and the subsurface layer that extends from 20 to 40 centimeters deep. The collected samples underwent a thorough screening process by passing them through a 5-mm nylon mesh. This step was essential to eliminate any unwanted materials, including roots, stones, and any visible plastic debris that could interfere with the analysis. By ensuring that the samples were free from such contaminants, the integrity of the data collected in subsequent analyses was maintained. Each sample was then divided into two subsamples: one was air-dried for the analysis of soil physicochemical properties and quantification of MPs, while the other was sifted through a 2-mm mesh, rapidly frozen in liquid nitrogen, and preserved at −80°C for microbial DNA extraction and high-throughput sequencing. During the sampling process, cotton lab coats and nitrile gloves were worn, and blank controls (which did not contain any soil samples) were included to mitigate potential MP contamination throughout the experimental procedures (Supplementary Figure 1).

### 2.2 Separation of MPs

The extraction procedure was adapted from Liu et al. (2018).

### 2.3 Observation and identification of MPs

A microscope was employed to detect MPs on filter paper, with their sizes, shapes, and classifications noted based on prior research. According to their morphological characteristics, the MPs were divided into four categories: films, fragments, fibers, and microbeads. Simultaneously, they were sorted into four size groups: 0–0.5 mm, 0.5–1.0 mm, 1.0–3.0 mm, and 3.0–5.0 mm. In this study, the abundance of MPs was reported as the number of items per 1 kg of dry soil, expressed in items kg^–1^. To confirm their polymer composition, all suspected microplastic particles classified based on morphology were further analyzed using Fourier-transform infrared spectroscopy (μ-FTIR, Spotlight 400, PerkinElmer, United States). Spectra were recorded in the range of 4,000–400 cm^–1^ at a resolution of 4 cm^–1^, with 32 scans accumulated per sample. The obtained spectra were compared with the OMNIC polymer standard spectral library for identification.

### 2.4 Soil physicochemical properties measurements

Soil moisture levels were determined by employing the oven drying method (Cui et al., 2020). A pH meter and a conductivity meter were utilized to assess the soil’s pH and electrical conductivity (EC), respectively, with soil-to-water ratios set at 1:2.5 (w/v) and 1:5 (w/v). For the analysis of ammonium nitrogen (NH_4_^+^-N) and nitrate nitrogen (NO_3_^–^-N) in the soil, a 2 mol⋅L^–1^ KCl solution was used at a soil-to-water ratio of 1:5 (w/v), with measurements performed using a flow analyzer. The total phosphorus (TP) and available phosphorus (AP) were measured using the molybdenum blue method in conjunction with an ultraviolet spectrophotometer. Soil organic carbon (SOC) and total nitrogen (TN) content quantification was achieved with an elemental analyzer (Elementar Vario MICRO cube, Hanau, Germany).

### 2.5 Extracellular enzymatic activity and quantification of microbial nutrient limitation

The β-1,4-glucosidase (BG), β-D-cellobiohydrolase (CBH), β-1,4-N-acetylglucosaminidase (NAG), leucine aminopeptidase (LAP) and alkaline phosphatase (ALP) activities were assessed using a biochemical assay kit.

Based on the original proportional activities [(BG + CBH)/(BG + CBH + NAG + LAP)], we determined the vectors’ lengths and angles for enzymatic activity across all data to assess microbial nutrient limitation. The vector length, an indicator of carbon limitation, was computed as the square root of the sum of the squares of x (the relative activities of enzymes acquiring C and P) and y (the relative activities of enzymes acquiring C and N)


$$L\,e\,n\,g\,t\,h=S\,Q\,R\,T\,\left(x^{2}+y^{2}\right)$$

The angle of the vector, indicating the N/P constraint, was determined by taking the arctangent of the line that extends from the origin of the plot to the coordinates (x, y):


Angle(°) = Degrees(Atan2 (x,y))

As the length of the vector increases, the limitation of microbial C also rises. When the vector angle exceeds 45°, it indicates a limitation of microbial P, while angles below 45° signify a limitation of microbial nitrogen (N).

### 2.6 Soil microbial DNA extraction, PCR amplification, and Illumina sequencing

Genomic DNA was isolated from soil samples using the cetyltrimethylammonium bromide (CTAB) method following the established protocol with modifications (Zhou et al., 1996). Briefly, approximately 0.5 g of soil was subjected to cell lysis in CTAB extraction buffer (100 mM Tris-HCl, 1.4 M NaCl, 20 mM EDTA, 2% CTAB, pH 8.0) at 65°C for 1 h. Proteins and other impurities were removed by chloroform-isoamyl alcohol (24:1) extraction. The DNA was then precipitated with isopropyl alcohol, washed with 70% ethanol, and finally dissolved in TE buffer (10 mM Tris-HCl, 1 mM EDTA, pH 8.0). The purified DNA was quantified using a Nanodrop 2000 spectrophotometer (ThermoFisher Scientific, Inc., United States) and adjusted to a working concentration of 50 ng μL^–1^ for subsequent PCR amplification. Specifically, the hypervariable regions V3–V4 of the bacterial 16S rRNA genes, which encompass approximately 420 base pairs, were amplified using universal primer pairs: 338F (ACTCCTACGGGAGGCAGCAG) and 806R (GGACTACHVGGGTWTCTAAT). In addition to this, the fungal internal transcribed spacer (ITS1) regions were amplified with a specific primer set comprised of ITS1F (CTTGGTCATTTAGAGGAAGTAA) and ITS2 (GCTGCGTTCTTCATCGATGC). Once the amplification was complete, all resulting products were sequenced using the Illumina Miseq platform provided by Biomarker Technologies, employing paired-end sequencing technology to ensure comprehensive data collection. The generated data from the 16S rRNA and ITS sequence were processed bioinformatically using the QIIME 2 pipeline. Through this sophisticated analysis, β-diversity indices were calculated, allowing for a quantitative comparison of taxonomic composition variations among the different experimental groups, thereby providing insights into the microbial community structures under investigation. In this study, stringent quality control and preprocessing were applied to the paired-end sequences generated from 16S rRNA and ITS sequencing. The specific pipeline included the following steps: First, raw sequences were subjected to quality filtering, during which regions with a sequencing error rate higher than 0.005% (corresponding to a Phred quality score > 45) were removed. Subsequently, sequence assembly was performed with a minimum overlap length of 10bp, and statistical testing with a *p*-value threshold of 0.0001 was employed to ensure assembly accuracy. Finally, chimeric sequences were detected and removed using a standard algorithm. These steps were carried out primarily with tools such as PEAR (v0.3.11) and VSEARCH (v2.27.0) to minimize sequencing errors and artificial artifacts, thereby ensuring high reliability of the sequence data used for subsequent microbial diversity and network analyses. To directly evaluate whether sequencing depth was sufficient and to ensure comparability among samples, rarefaction curves were generated for both bacterial and fungal communities. As shown in Supplementary Figure 2, all sample curves reached a clear plateau before the sequence count used in the current analysis (i.e., the rarefaction depth), indicating that the sequencing effort was adequate to capture the majority of microbial diversity present in the samples.

### 2.7 Statistical analysis

Microsoft Excel 2021 was utilized to conduct statistical analyses on basic chemical data and species classification data. Additionally, SPSS 26.0 (IBM Crop, Chicago, IL, United States) was employed for variance analysis (ANOVA), correlation analysis (Pearson) and Duncan’s post hoc test for multiple comparisons (*P* < 0.05). Statistical analyses were conducted using R (Version 4.4.0), where the Psych package (Huang et al., 2023) identified significant correlations (|*r*| > 0.8, *p* < 0.05) for co-occurrence network construction. Network visualization was implemented in Gephi 0.9.2, with topological parameters quantified through the igraph package. Mantel test analysis was performed using the ggcor R package to assess variable associations. To systematically disentangle direct and indirect relationships among mulching duration, MP attributes, soil properties, microbial composition, and nutrient limitations, a structural equation model (SEM) was developed in Amos (Version 23.0). Microbial community dimensionality reduction was executed via the SPSPRO cloud-based analytical platform. Model adequacy was assessed using three key metrics: non-significant chi-square statistic (*p* > 0.05), acceptable CMIN/DF ratio (<3), and comparative fit index (CFI > 0.9).

## 3 Results

### 3.1 Abundances, size, shapes, and polymer composition of MPs in soil under different mulching years

**FIGURE 1:**
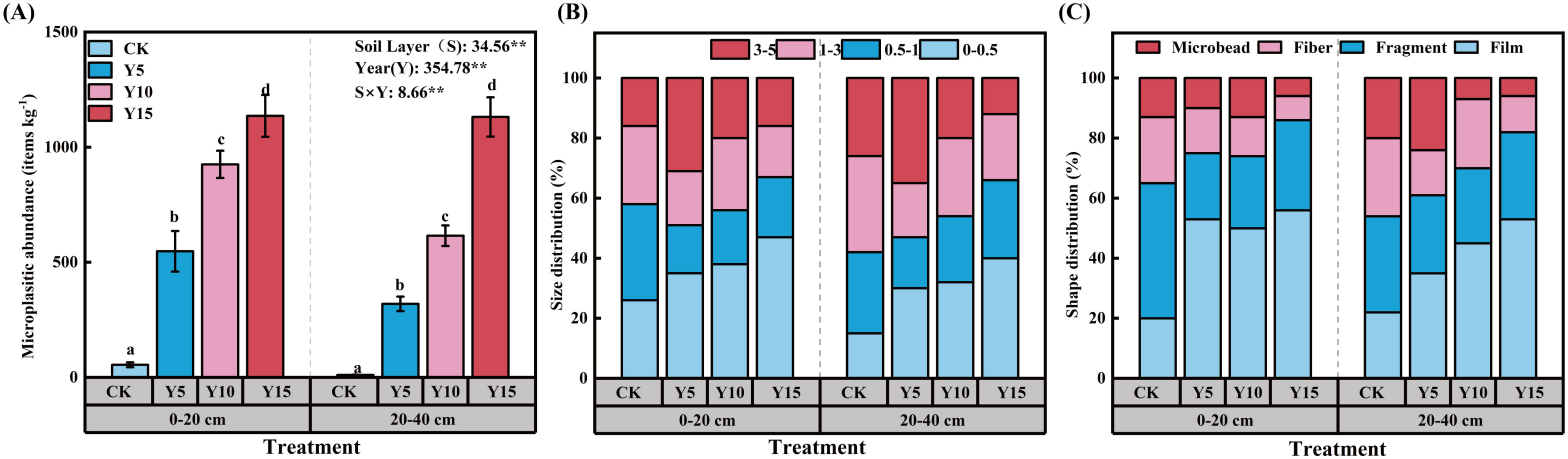
Abundance **(A)**, type distribution **(B)** and size distribution **(C)** of MPs collected under different mulching years. Error bars indicate standard error; *p* values are based on one-way ANOVA. Different lowercase letters indicate significant differences at *p* < 0.05 among treatments. **Represents significantly different at the 0.01 level. *Represents significantly different at the 0.05 level; ns represents no significant difference.

The results indicated that MPs were detected in all soil samples, with significant differences in abundance across samples with different film mulching years and soil depths (*P* < 0.05). The abundance of MPs ranged from 10.67 to 1136.00 pieces kg^–1^, with a notable trend showing that longer film covering times correlated with higher MP accumulation. In comparison to the non-film covering treatment, the abundance of MPs in surface soil increased by factors of 4.8, 10.3, and 19.7 after 5, 10, and 15 years of film mulching, respectively. A similar trend was also observed in the subsoil (Figure 1A).

Based on the size characteristics of MPs, they were categorized into four groups: 0–0.5, 0.5–1, 1–3, and 3–5 mm. Generally, MPs in the 0–0.5 mm category were predominant across all samples, comprising 15–42% of the total. The proportions of the other three size categories did not differ significantly, an increase in mulching year was associated with a marked rise in the proportion of small-sized MPs (0–0.5 mm). Conversely, the proportion of larger MPs (3–5 mm) gradually decreased, with consistent trends observed in both soil layers (Figure 1B).

Based on the morphological characteristics of MPs, those found in continuously mulched maizefields can be categorized into four types: films, fragments, fibers, and microbead. Among these, films and fragments are the predominant forms observed, accounting for 20–56 and 22–45%, respectively, in the surface soil layer; in the subsurface soil layer, they account for 22–45 and 25–32%, respectively. The fiber and microbead types are significantly fewer in proportion compared to films and fragments (Figure 1C).

FTIR spectroscopy revealed the diversity of polymer types among the soil microplastics. Polyethylene was identified as the PE, accounting for 95.3% of all detected particles. Polypropylene (PP) and polyethylene terephthalate (PET, commonly referred to as polyester fiber) were also detected across all treatment groups, together representing an average of 4.7% of the total microplastic content. (Supplementary Figures 3, 4)

### 3.2 Soil physicochemical properties under different mulching years

Different soil depths significantly affect water content, SOC, TN, NO_3_^–^-N, TP, and AP (*P* < 0.01). As soil depth increases, soil water content also increases, with the lower layer showing an 11.45% increase compared to the surface soil. Conversely, EC, SOC, TN, NO_3_^–^-N, TP, and AP all decrease with increasing soil depth. Furthermore, the physical and chemical properties exhibit significant variation across different mulching years (*P* < 0.05). With an increase in mulching years, soil moisture content, EC, TP, and AP demonstrate an upward trend, while SOC, TN, NO_3_^–^-N, and NH_4_^+^-N exhibit a downward trend, with consistent changes observed between the two soil layers (Table 1).

**TABLE 1:** Soil physicochemical properties under different mulching years.

Soil layer	Year	Water content (%)	EC (us/cm)	SOC (g kg^–1^)	TN (g kg^–1^)	NO_3_^–^-N (mg kg^–1^)	NH_4_^+^-N (mg kg^–1^)	TP (g kg^–1^)	AP (mg kg^–1^)
0–20 cm	CK	16.0 ± 1.4 a	88.9 ± 10.9 b	18.9 ± 0.3 a	1.3 ± 0.1 a	55.5 ± 12.1 a	6.1 ± 0.2 a	0.9 ± 0.0 d	9.0 ± 1.3 c 12.7 ± 0.9 b 13.6 ± 0.8 b 16.3 ± 0.7 a **12.9 ± 2.8 A**
	Y5	15.9 ± 0.8 a	101.6 ± 2.3 ab	15.2 ± 0.1 b	1.0 ± 0.2 ab	51.5 ± 0.9 a	5.0 ± 0.4 ab	0.7 ± 0.0 c	
	Y10	17.1 ± 1.8 a	104.3 ± 13.9 ab	13.9 ± 0.6 c	0.9 ± 0.1 b	50.0 ± 4.1 a	3.7 ± 1.4 bc	1.2 ± 0.1 b	
	Y15	17.2 ± 1.9 a	116.3 ± 9.4 ab	13.9 ± 0.3 c	0.9 ± 0.0 b	43.7 ± 0.4 a	3.3 ± 0.4 c	1.5 ± 0.1 a	
	**Overall mean**	**16.6 ± 1.5 B**	**102.8 ± 13.3 A**	**15.5 ± 2.1 A**	**1.0 ± 0.2 A**	**50.2 ± 7.0 A**	**4.5 ± 1.3 A**	**1.1 ± 0.3 A**	
20–40 cm	CK	14.7 ± 2.7 b	94.6 ± 5.0 a	14.2 ± 1.0 a	0.9 ± 0.0 a	54.0 ± 2.7 a	5.9 ± 0.7 a	0.7 ± 0.0 c	7.7 ± 1.2 b 5.7 ± 0.4 b 12.8 ± 3.0 a 11.4 ± 1.2 a **9.4 ± 3.3 B**
	Y5	16.8 ± 0.6 b	104.6 ± 1.9 a	13.9 ± 0.3 ab	0.9 ± 0.1 ab	43.4 ± 5.7 b	4.0 ± 0.8 b	0.5 ± 0.0 b	
	Y10	21.4 ± 1.3 a	107.9 ± 17.6 a	12.6 ± 1.3 ab	0.8 ± 0.1 ab	42.3 ± 1.6 b	3.5 ± 0.4 bc	0.9 ± 0.0 a	
	Y15	21.2 ± 1.9 a	108.3 ± 12.3 a	12.4 ± 0.5 b	0.7 ± 0.2 b	33.7 ± 0.5 c	2.8 ± 0.4 c	0.9 ± 0.0 a	
	**Overall Mean**	**18.5 ± 3.4 A**	**103.9 ± 11.0 A**	**13.3 ± 1.1 B**	**0.8 ± 0.1 B**	**43.4 ± 8.0 B**	**4.1 ± 1.3 A**	**0.7 ± 0.2 B**	
ANOVA	Soil Layer (S)	18.91**	0.065*^ns^*	63.58**	188.71**	10.78**	2.54*^ns^*	188.71**	37.40** 23.81** 6.66**
	Year (Y)	8.89**	3.98*	33.11**	117.99**	10.01**	21.91**	117.99**	
	S*Y	0.83^ns^	0.52^ns^	8.71**	16.90**	0.79^ns^	0.42^ns^	16.90**	

Values followed by different small letters within a column are significantly different at 5% probability level. **Represents significantly different at the 0.01 level. *Represents significantly different at 0.05 level; ns represents no significant difference. “Overall mean” refers to the overall mean of the data presented above. EC, electrical conductivity; SOC, soil organic C; TN, total nitrogen; TP, total phosphorus; AP, available phosphorous.

### 3.3 Activities and stoichiometric characteristics of soil carbon, nitrogen, and phosphorus extracellular enzymes under different mulching years

Soil extracellular enzyme activity and its measurement ratios are influenced by soil depth, the duration of film covering, and their interaction (*P* < 0.05). As soil depth increases, the activities of C, N, and P acquisition enzymes all decline. Specifically, compared to surface soil, the activities of these enzymes in deeper soil decreased by 36.4, 30.5, 22.4, 16.7 and 10.2%, respectively. Under mulching conditions, the activities of C and N acquisition enzymes are reduced by 36.5, 37.0, 38.4 and 16.2%, respectively, when compared to non-mulched soil. Conversely, the activity of P acquisition enzymes increases by 3.6% relative to non-mulched soil. Additionally, enzyme activity exhibits a decreasing trend with an increase in the number of mulching years, reaching its lowest value after 15 years of mulching. The ratios (BG + CBH)/(NAG + LAP), (BG + CBH)/ALP, and (NAG + LAP)/ALP are all significantly affected by soil depth and the years of film mulching (*P* < 0.01) (Table 2).

**TABLE 2:** The characteristics of enzyme activities and their stoichiometry under different mulching years.

Soil layer	Treatment	BG	CBH	NAG	LAP	ALP	(BG+CBH)/ (NAG+LAP)	(BG+CBH)/ ALP	(NAG+LAP)/ ALP
0–20 cm	CK	265.5 ± 15.1 a	29.5 ± 1.5 a	35.2 ± 1.0 a	147.7 ± 5.3 a	287.5 ± 14.4 c	1.6 ± 0.1 a	1.0 ± 0.1 a	0.6 ± 0.0 a 0.5 ± 0.0 b 0.5 ± 0.0 b 0.3 ± 0.0 c **0.5 ± 0.1 A**
	Y5	233.9 ± 9.3 b	23.9 ± 2.5 b	27.6 ± 2.0 b	133.6 ± 2.6 b	344.9 ± 10.2 b	1.6 ± 0.1 b	0.7 ± 0.0 b	
	Y10	210.6 ± 8.8 c	26.3 ± 1.1 b	25.5 ± 6.8 b	128.6 ± 12.6 b	333.5 ± 8.5 b	1.5 ± 0.2 bc	0.7 ± 0.0 b	
	Y15	130.1 ± 7.2 d	15.3 ± 2.5 c	13.1 ± 1.2 c	88.2 ± 2.6 c	365.9 ± 3.4 a	1.4 ± 0.1 c	0.4 ± 0.0 c	
	**Overall Mean**	**210.0 ± 57.8 A**	**23.8 ± 6.1 A**	**25.4 ± 9.2 A**	**124.5 ± 25.5 A**	**333.0 ± 33.1 A**	**1.5 ± 0.1 A**	**0.7 ± 0.3 A**	
20−⁣−40 cm	CK	207.6 ± 11.9 a	26.3 ± 3.0 a	28.1 ± 1.6 a	112.1 ± 5.5 a	327.7 ± 15.1 a	1.7 ± 0.1 a	0.7 ± 0.1 a	0.4 ± 0.0 ab 0.4 ± 0.0 c 0.4 ± 0.0 a 0.4 ± 0.0 b **0.4 ± 0.0 A**
	Y5	114.2 ± 5.6 b	17.4 ± 2.3 b	16.7 ± 2.4 c	82.3 ± 5.0 c	274.4 ± 9.2 c	1.3 ± 0.1 b	0.5 ± 0.0 b	
	Y10	120.9 ± 9.9 b	12.0 ± 0.9 c	13.6 ± 2.0 d	114.8 ± 2.8 a	288.3 ± 15.2 bc	1.0 ± 0.1 c	0.5 ± 0.1 b	
	Y15	91.6 ± 2.7 c	10.4 ± 0.8 c	20.5 ± 1.4 b	105.5 ± 2.5 b	305.9 ± 12.9 b	0.8 ± 0.0 d	0.3 ± 0.0 c	
	**Overall Mean**	**133.5 ± 50.9 B**	**16.5 ± 7.2 B**	**19.7 ± 6.3 B**	**103.7 ± 14.8 B**	**299.1 ± 23.0 B**	**1.2 ± 0.4 B**	**0.5 ± 0.2 B**	
ANOVA	Soil Layer (S)	497.17**	95.08**	33.49*	77.83**	78.4**	72.41**	197.82**	28.80** 65.33** 61.28**
	Year (Y)	234.74**	77.96**	45.07**	32.60**	10.13**	35.46**	182.08**	
	S*Y	30.12**	9.06**	18.25**	36.64**	41.92**	15.19**	16.26**	

Each value represents the mean ± standard error. Values followed by different small letters within a column are signiffcantly different at 5% probability level. *Represents signiffcantly different at 0.05 level. **Represents signiffcantly different at the 0.01 level; ns represents no significant difference. “Overall mean” refers to the overall mean of the data presented above. BG, β-1, 4-glucosidase; CBH, β-D-cellobiohydrolase; NAG, β-1, 4-N-acetylglucosaminidase; LAP, leucine aminopeptidase; ALP, alkaline phosphatase.

### 3.4 Soil microbial nutrient limitation vector analysis under different mulching years

**FIGURE 2:**
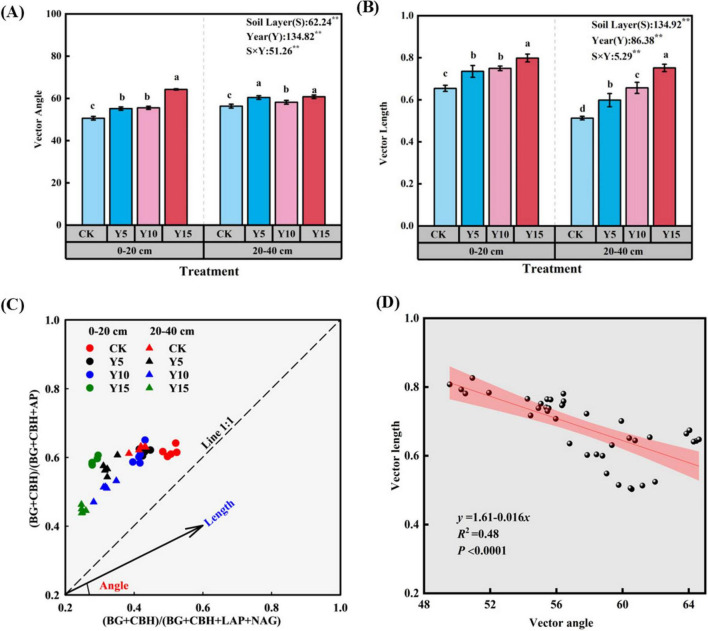
Vector angle **(A)**, vector length **(B)**, scatter plot of the microbial resource limitation distribution **(C)** and relationships between the vector length and vector angle under different mulching years **(D)**.

By calculating the vector length and angle, we quantified the relative C and P limitations of microorganisms, with vector lengths ranging from 0.51 to 0.79 and vector angles from 50.6 to 64.2 degrees. These parameters exhibited substantial changes with variations in soil depth and mulching time (*P* < 0.01). As soil depth increased, the vector length decreased, showing a reduction of 14.2% compared to surface soil, while the vector angle increased by 4.5% relative to surface soil. With an increase in mulching time, the vector length and vector angle displayed an upward trend (Figures 2A,B). The stoichiometric characteristics of soil enzymes varied with soil depth and the duration of film mulching. All data points were positioned above the 1:1 line, indicating that the soil microbial community in the study area was significantly limited by P (Figure 2C). Moreover, a linear regression analysis indicated a strong negative correlation (*P* < 0.001) between vector length and angle (Figure 2D).

### 3.5 Soil microbial diversity and abundance under different mulching years

Bacteria and fungi are significantly influenced by mulching time. In the surface soil, the mulching treatment significantly enhanced both the Chao1 index and the Shannon index of the bacterial community, with increases of 7.8 and 4.2%, respectively, compared to the no-mulching condition. The most pronounced increase was observed in the treatment with mulching film applied for 10 years, followed by those with 5 years and 15 years of mulching. In the deep soil, only the 10-year mulching treatment resulted in a significant increase in the bacterial Chao1 and Shannon indices, while other treatments did not show significant changes. For the fungal community, in the surface soil, the mulching treatment significantly raised the Chao1 index, with the most substantial increase occurring after 10 years of mulching. Conversely, in the deep soil, the mulching treatment led to reductions in both the Chao1 index and the Shannon index, with the 5- and 10-year treatments exhibiting the most significant declines (Table 3).

**TABLE 3:** The diversities of soil bacterial and fungal diversity under different mulching years.

Soil layer	Treatment	Bacterial community			Fungi community		
		Chao1 index	Shannon index	Simpson	Chao1 index	Shannon index	Simpson
0–20 cm	CK	4576.0 ± 607.8 ab	9.5 ± 0.4 ab	1.0 ± 0.0 a	499.9 ± 164.9 a	5.8 ± 1.6 a	0.9 ± 0.2 a
	Y5	4803.6 ± 656.6 a	9.8 ± 0.5 a	1.0 ± 0.0 a	527.5 ± 183.2 a	5.8 ± 0.6 a	0.9 ± 0.0 a
	Y10	5209.3 ± 21.3 ab	10.1 ± 0.1 ab	1.0 ± 0.0 a	647.0 ± 97.4 a	5.1 ± 0.7 a	0.9 ± 0.1 a
	Y15	4780.9 ± 357.5 ab	9.8 ± 0.5 ab	1.0 ± 0.0 a	533.7 ± 82.1 a	5.0 ± 0.4 a	0.9 ± 0.1 a
	**Overall Mean**	**4842.5 ± 506.9 A**	**9.8 ± 0.4 A**	**1.0 ± 0.0 A**	**552.0 ± 141.6 A**	**5.4 ± 0.9 A**	**0.9 ± 0.1 A**
20–40 cm	CK	4775.4 ± 325.6 ab	9.7 ± 0.2 ab	1.0 ± 0.0 a	564.7 ± 69.6 a	5.9 ± 1.3 a	0.9 ± 0.1 a
	Y5	4316.8 ± 477.6 b	9.4 ± 0.3 b	1.0 ± 0.0 a	363.1 ± 83.2 b	5.7 ± 0.4 a	1.0 ± 0.0 a
	Y10	5036.5 ± 302.4 a	10.0 ± 0.3 a	1.0 ± 0.0 a	478.8 ± 190.9 ab	4.9 ± 0.8 a	0.9 ± 0.1 a
	Y15	4757.3 ± 366.7 ab	9.8 ± 0.4 ab	1.0 ± 0.0 a	505.4 ± 120.3 ab	5.3 ± 0.6 a	0.9 ± 0.1 a
	**Overall Mean**	4721.5 ± 437.5 A	**9.7 ± 0.4 A**	**1.0 ± 0.0 A**	**478.0 ± 138.7 A**	**5.4 ± 0.9 A**	**0.9 ± 0.1 A**
ANOVA	Soil Layer (S)	0.94^ns^	0.85^ns^	0.20^ns^	3.77^ns^	0.00^ns^	0.13^ns^
	Year (Y)	3.80*	3.65*	0.73^ns^	1.71^ns^	3.08*	1.35^ns^
	S*Y	0.34^ns^	1.60^ns^	0.20^ns^	2.20^ns^	0.17^ns^	0.06^ns^

Each value represents the mean ± standard error. Values followed by different small letters within a column are signiffcantly different at 5% probability level. *Represents signiffcantly different at 0.05 level. ns represents no significant difference. “Overall mean” refers to the overall mean of the data presented above.

**FIGURE 3:**
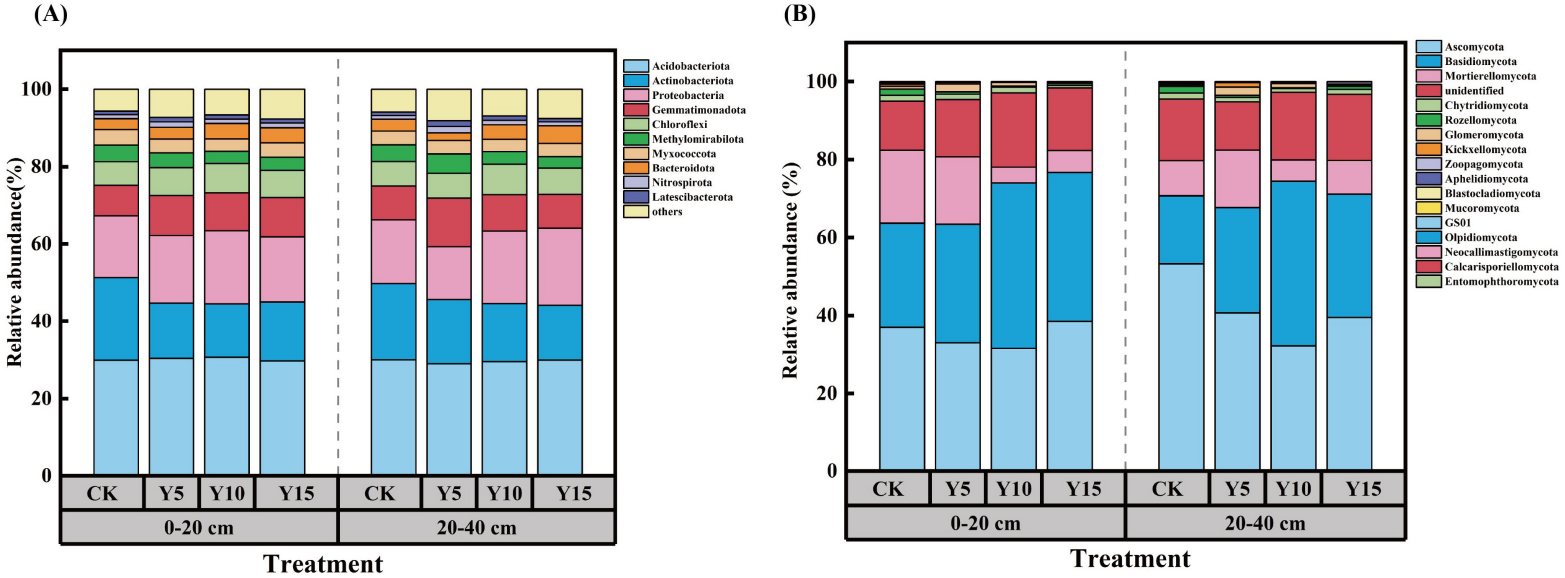
Relative abundance of soil bacteria at phylum under different mulching years **(A)**; relative abundance of soil fungi at phylum under different mulching years **(B)**.

The relative abundance of soil microorganisms differed significantly at the phylum level between different years of mulching. The most prevalent bacterial phyla were identified as *Acidobacteriota* (29.53–30.68%), *Actinobacteriota* (13.85–21.41%), *Proteobacteria* (13.62–19.89%), *Gemmatimonadota* (7.85–12.51%) and *Chloroflexi* (6.17–7.98%). The relative abundance of the *Actinobacteriota* and *Gemmatimonadota* exhibited significant variation following mulching, with a substantial increase in *Actinobacteriota* abundance and a concomitant decrease in *Gemmatimonadota* abundance being observed (Figure 3A). This trend was consistent across both soil layers. The most prevalent phyla for fungi were *Ascomycota* (31.61–53.29%), *Basidiomycota* (17.41–42.36%), *Mortierellomycota* (4.06–18.67%) and *Chytridiomycota* (0.69–1.48%) (Figure 3B). Mulching resulted in a significant increase in the relative abundance of B*asidiomycota* compared to non-mulched soil.

### 3.6 Soil microbial co-occurrence network analysis under different mulching years

**FIGURE 4:**
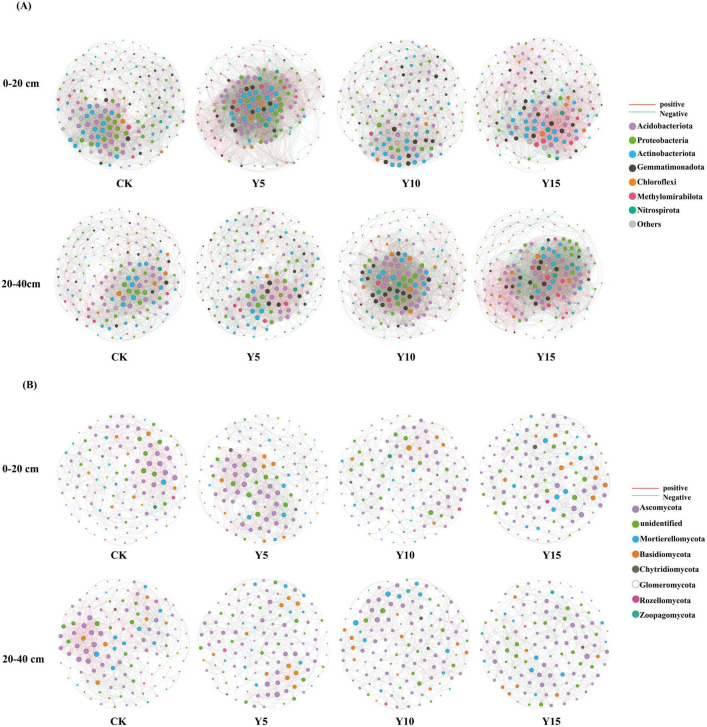
Bacteria **(A)** and fungi **(B)** association networks under different film mulching (phylum level).

The diagram of the symbiotic network demonstrates that in the surface soil, the application of film mulching substantially enhanced the number of edges, average degree, average clustering coefficient, network diameter, and network density of the bacterial network, all while concurrently decreasing the average path length. After 5 years of film mulching, the increases in the number of network edges, degrees of freedom, and network density were most pronounced. Modularity reached its highest levels after mulching 10 years, with the latter displaying the strongest positive correlation after mulching 15 years. In the deep soil, the film coating treatment similarly enhanced the number of edges, average clustering coefficient, average degree, network diameter, and network density, while decreasing both positive correlation and average path length. Among these changes, the most significant increase in the number of edges after mulching 10 and 15 years, and network modularity increased significantly after 5 years of lamination (Figure 4A; Supplementary Table 2).

In the surface soil, the film mulching treatment reduced the positive correlation, average path length, and network diameter of the fungal network, while increasing the negative network correlation, average clustering coefficient, average degree, and network density. In comparison to various mulching film time, a 5-year application of mulching film increased the number of network edges. A 10-year mulching significantly enhanced network modularity, while a 15-year application notably increased the negative correlation within the network. In the deep soil, the film mulching treatment resulted in a reduction of the average clustering coefficient, average degree, network diameter, and network density of the network edges, while simultaneously increasing the negative correlation and network path length. The changing trends across different mulching years were generally consistent (Figure 4B; Supplementary Table 3).

### 3.7 The relationship between soil physicochemical properties and MPs traits

Through the correlation heat map, we can see that microplastic abundance has a highly significant negative correlation with SOC, NO_3_^–^-N, NH_4_^+^-N, TN, and Vector Length, and a highly significant positive correlation with soil water content, EC, AP, TP, and Vector Angle; for microplastic morphology, thin film-type MPs have the greatest impact on soil physicochemical properties, and with SOC, NO_3_^–^-N, NH_4_^+^-N, TN, and Vector Length, and was significantly positively correlated with soil water content, EC, AP, TP, and Vector Angle; for MPs with different particle sizes, 0–0.5 mm MPs were the main type of MPs affecting the soil physicochemical properties, and were significantly negatively correlated with NO_3_^–^-N, NH_4_^+^-N, and Vector Length, and EC, AP, TP, and Vector Angle (Supplementary Figure 5).

### 3.8 Relationships among soil chemical properties, stoichiometry, and microbial communities

The Mantel test was conducted on the microbial community and soil physicochemical properties and stoichiometry. In the 0–20 cm soil layer, the bacterial community was most strongly correlated with SOC, NH_4_^+^-N, AP, TP, Vector length and Vector angle (*P* < 0.05), and the fungal community was most strongly correlated with SOC, NH_4_^+^-N and AP (*P* < 0.05) (Supplementary Figure 6A); in the 20–40 cm soil layer, the bacterial community was only correlated with TP and AP, and the fungal community was correlated with NO_3_^–^-N and soil water content (Supplementary Figure 6B).

### 3.9 Correlation analysis of environmental factors, MP characteristics and nutrient limitations

**FIGURE 5:**
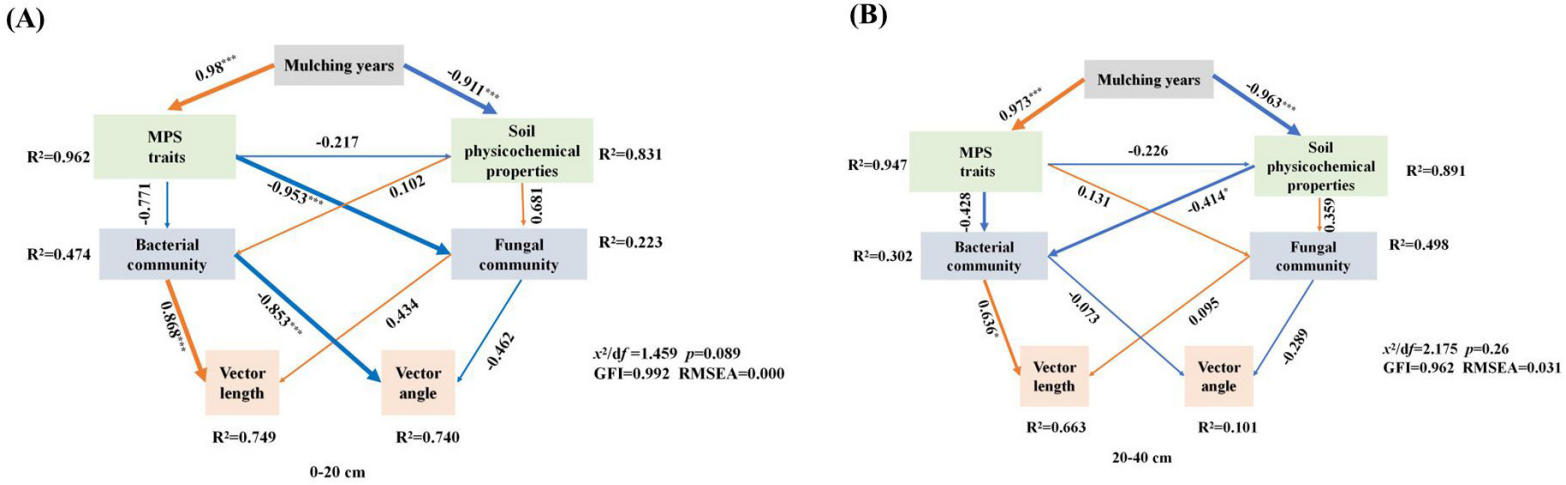
Structural equation model was used to analyze the direct and indirect relationships between film mulching years and microplastic traits, soil physicochemical properties, bacterial communities, fungal communities and C, N nutrient limits (**A:** 0–20 cm; **B:** 20–40 cm). The arrow width is proportional to the strength of the path coefficients. Solid red arrows indicate a positive correlation, while blue dotted arrows indicate a negative relationship. * and *** indicate *P* < 0.05 and *P* < 0.001, respectively.

Structural Equation Modeling (SEM) was utilized to examine the complex interactions between the duration of mulching film, properties of MPs, soil physical and chemical characteristics, and bacterial and fungal communities. The findings reveal that the length of time using mulching significantly altered the attributes of MPs (leading to higher abundance and smaller sizes), as indicated by a path coefficient surpassing 0.9 (*P* < 0.0001). In contrast, it negatively affects soil characteristics, with a path coefficient also exceeding 0.9 (*P* < 0.0001). Additionally, the communities of bacteria and fungi are demonstrated to indirectly influence these impacts, affecting microbial nutrient limitations. Importantly, both bacteria and fungi are found to adversely affect the VA while positively influencing the VL (Figure 5).

## 4 Discussion

### 4.1 Traits of MPs under different mulching years

Research has indicated that the global average abundance of soil MPs in plastic film mulching is approximately 158 pieces kg^–1^ (Büks and Kaupenjohann, 2020). However, our research revealed that this value is significantly higher than the global average concentration (Figure 1A; Sajjad et al., 2022; Yu et al., 2023). As the duration of mulching increases, a considerable amount of residual mulch film weathers and degrades into MPs, gradually accumulating in the farmland soil. The cumulative effect of extended mulching periods becomes increasingly pronounced. This study reveals that the abundance of MPs in surface soil is significantly higher than in deeper soil layers (Figure 1A). Several factors contribute to this phenomenon. Firstly, residual mulch films are primarily concentrated on the soil surface, resulting in a greater abundance of MPs in the upper soil layers. Additionally, agricultural practices, such as plowing prior to crop sowing, facilitate the downward movement of MPs from the upper soil to deeper layers. The extent of this migration is exacerbated by the duration of tillage (Zhao et al., 2022). Consequently, significant differences in MP abundance values are observed between the various soil layers. Secondly, due to their small size, MPs are susceptible to downward migration through soil pores, facilitated by rainwater and drip irrigation (Xu et al., 2024). Lastly, soil animals, such as earthworms, may ingest MPs or have them adhere to their surfaces, thereby expanding the migration range as these organisms move through the soil (Chang et al., 2022). This study found that most MPs were less than 3 mm in diameter, with an increasing number of small-sized MPs correlating with the time of mulching (Figure 1B). As the duration of the mulch film increases, the residual film experiences various factors like UV exposure, thermal degradation, physical wear, and biodegradation, which gradually lead to its breakdown into smaller MPs over time (Golmohammadi et al., 2023). Moreover, additional research has suggested that fertilization could expedite the breakdown of plastic debris. Research indicates that the application of N or P fertilizers enhances the diversity and population of specific bacteria or fungi that play a role in breaking down MPs, subsequently leading to a significant increase in the quantity of smaller MP fragments (Zhang et al., 2020). In this experiment, the types of MPs identified in the soil included films, fragments, fibers, and microbead (Figure 1C). Films and fragments were the predominant types, while fibers and microbead were less common. These mulching films have weathered and degraded into films and fragments, which subsequently decomposed into fibers and granules. However, due to the fact that most mulching films are composed of polyethylene and possess stable chemical properties, this degradation process can take decades or even centuries. As a result, thin films and fragments constitute a significant proportion of the MPs present in the soil (Clinton and Rowe, 2024). The PE identified in this study is directly linked to the long-term agricultural practice of applying LDPE plastic film locally, with its fragmentation process serving as the primary initial source of microplastics in the surface soil. This finding aligns with previous research on mulched farmland. Additionally, the detection of PET and PP indicates parallel input pathways of microplastics beyond plastic film. These may originate from: (1) textile fibers shed during laundry in domestic wastewater, which constitute a major component of microplastics in sludge; (2) the aging and fragmentation of agricultural textiles (e.g., shading nets, insect-proof nets); and (3) long-distance transport via atmospheric dry/wet deposition or irrigation water (Huang et al., 2020).

### 4.2 Effect of MPs on soil physicochemical properties under different mulching years

This study reveals that with an increase in the duration of mulching, soil moisture content and electrical conductivity rise, while SOC, TN, NO_3_^–^-N, and NH_4_^+^-N levels exhibit a declining trend (Table 1). The associated analysis heat map and structural equation model further demonstrate the negative impact of mulching film on soil physical and chemical properties (Supplementary Figure 5, 6). Additionally, properties related to MPs were found to be negatively correlated with these soil characteristics. Continuous mulching not only diminishes soil nutrient content but also introduces MPs that adversely affect soil nutrients. Research indicates that MPs particles may create physical barriers within the soil, hindering the infiltration of water and air, which in turn affects soil aeration and drainage. This disruption can indirectly influence nutrient cycling and microbial activity within the soil (Mondol et al., 2024; Sajjad et al., 2022). Long-term film mulching leads to the mineralization of organic carbon in soil aggregates and a significant reduction in SOC, akin to the observed decrease in organic carbon levels in soil aggregates following extended film mulching (Zhou et al., 2020). Additionally, long-term mulching enhances soil moisture and temperature, thereby promoting the development of plant root systems, increasing root secretions, and improving the nutrient utilization efficiency of the soil. However, the application of mulch introduces MPs into the soil, which can alter the composition and function of the microbial community, facilitate the decomposition of organic matter, and negatively impact soil microbial biomass. Other studies indicate that MPs generated from film mulching may enhance the metabolic efficiency of microorganisms and accelerate the decomposition of SOC by improving soil aeration (Zhang Y. et al., 2022).

### 4.3 Effect of MPs on soil microbial communities under different mulching years

In this experiment, we observed an increase in the diversity of bacteria and fungi in the surface soil following mulching (Table 3). This increase may be attributed to the substantial accumulation of film debris in the soil due to long-term mulching (Zhao et al., 2024; Ma et al., 2025). For bacteria, mulching increased the abundance of *Acidobacteriota*, which are mostly hygrophilous, and mulching provides suitable conditions for their survival (Yang et al., 2021). At the same time, *Acidobacteriota* contain facultative anaerobe, which survive and reproduce through fermentation or reductive metabolism under low-oxygen conditions, and MPs produced by mulching will also directly drive *Acidobacteriota* enrichment (Qiu et al., 2023). MPs from mulching also directly drive the enrichment of *Acidobacteriota*, some strains of *Acidobacteriota* carry plastic degradation genes that slowly degrade PE to release oligomers (Qiu et al., 2023). Moisture saturation will significantly reduce the oxygen content in the soil pore space, making the soil environment tend to anaerobic, most of the known *Gemmatimonadota* members are aerobic or partially aerobic bacteria (Mujakić et al., 2022), and they prefer a well-oxygenated soil environment for respiration, and at the same time, the increase of MPs after mulching, affecting the connectivity of the soil pore space further exacerbates the problem of soil hypoxia, which directly inhibits their growth and activities, and reduces their abundance. At the same time, the rapid mineralization depletion of readily decomposable organic matter may lead to a decline in soil organic matter quality at the later stage, which may also be detrimental to the survival of some *Gemmatimonadota* taxa. The increased abundance of *Basidiomycota* in the fungal community was mainly related to the following changes in the physical, chemical and biological environment of the soil caused by mulching, which reduces the evaporation of soil water and maintains relatively stable and high soil moisture (Zhang et al., 2021). *Basidiomycota* usually require higher moisture activity for growth and reproduction (e.g., mycelial extension, substrate formation), and the moisture conditions created by mulching are and the moisture conditions created by mulching are more favorable to their activities. Certain *Basidiomycota* species (especially some white-rot fungi) are naturally more tolerant to environmental stresses. They may have a greater tolerance or detoxification mechanism to additives (e.g., certain plasticizers) released from MPs or adsorbed pollutants (e.g., heavy metals, organic pollutants) than bacteria or other fungal phyla.

The enhancement of microbial network complexity and stability, as evidenced by the increase in nodes, edges, and average degree, suggests that the ecosystem exhibits greater resilience and is better equipped to withstand environmental disturbances. Previous research on variations in microbial network structures across different habitat types has demonstrated that network complexity increases with higher precipitation levels. This study, utilizing co-occurrence network analysis, reveals the reshaping effect of long-term plastic film mulching on soil microbial interaction relationships. The findings indicate that under mulching conditions, bacterial networks show a significant increase in complexity (in terms of edge number, average degree, and network density), highlighting enhanced microbial interactions, particularly the strengthening of synergistic relationships in the surface soil. The formation of this network structure may arise from the heterogeneous microhabitats generated by the accumulation of MPs, which create diverse ecological niches for bacteria, thereby promoting functional redundancy and community stability. The notable increase in bacterial network modularity after 5 years of mulching suggests that microorganisms adapt to resource pressures through a strategy of functional partitioning-exhibiting tight cooperation within modules to efficiently utilize limited resources while minimizing competitive consumption between modules. This phenomenon is linked to the observed increase in bacterial diversity and the rise in *Actinobacteriota* abundance. In contrast, the fungal network exhibited contradictory responses to the mulching film. In the surface soil, negative correlations intensified, reflecting heightened interspecific resource competition. This is likely associated with the competitive utilization of recalcitrant organic matter by fungi in the carbon-deficient environment induced by MPs. Conversely, in the deeper soil, the network density and connectivity declined (Supplementary Table 3), indicating a reduced degree of niche differentiation. This phenomenon aligns with the decreased diversity of the fungal community, where the increased relative abundance of *Basidiomycota* suggests that certain tolerant taxa, such as those capable of degrading pollutants adsorbed by MPs, may dominate the community structure through competitive advantages, ultimately leading to a simplification of the interaction network.

### 4.4 Effect of MPs on soil microorganism nutrient limitations under different mulching years

MPs in soil can significantly reduce soil enzyme activity and microbial abundance (Yu et al., 2020). In this study, a decreasing trend in the activities of carbon- and nitrogen-acquiring enzymes was observed with increasing mulching duration (Table 2), a result that may be attributed to comprehensive changes in the soil environment. The increases in soil moisture and EC (Table 1) likely altered soil pore aeration and osmotic conditions, thereby affecting the activity of aerobic microorganisms as well as the synthesis and secretion of enzymes. Simultaneously, the significant decreases in SOC and TN (Table 1) limited the substrate supply for related enzymatic reactions, which may have directly constrained the synthesis and catalytic efficiency of enzymes involved in C and N cycling. Therefore, the changes in enzyme activity reflect not only the accumulation effect of microplastics but also the integrated influence of concomitant variations in soil moisture and nutrient conditions (Ren et al., 2020). The presence of MPs interferes with these processes through various mechanisms, including physical barriers, chemical adsorption, and biological toxicity, inhibiting interactions with carbon and nitrogen. Extracellular enzyme activities closely associated with the phosphorus cycle-such as cellulase, urease, and phosphatase are also affected, ultimately slowing the decomposition of organic matter and the release rate of nutrients (Liu et al., 2024). The toxic effects of MPs extend beyond direct physical and chemical damage; they can also induce microbial cell dysfunction, protein denaturation, and cell membrane damage. These physiological injuries compel microorganisms to increase substrate consumption in response to toxic pressure in order to synthesize antioxidant substances and sustain basic life activities (Wang W. et al., 2022; Ji et al., 2023). During this process, the demand for carbon and phosphorus sources among microorganisms rises sharply, particularly when confronted with nanoplastics (NPs) resulting from the decomposition of MPs. Due to their small size and high specific surface area, NPs can adsorb and release toxic substances more effectively, further exacerbating the nutritional stress experienced by microorganisms. MPs enhance the breakdown of litter and soil organic matter by altering the structure and functionality of microbial communities (Zhou X. et al., 2024). This change may lead to an increased rate of soil respiration, resulting in the swift depletion of soil carbon reserves and causing significant microbial carbon limitations. The escalation of these limitations further disrupts phosphorus cycling and its availability. Phosphorus is vital for both plant development and microbial processes, and its presence and form in the soil are greatly affected by the decomposition of organic matter (Cao et al., 2024). Vector analysis of enzyme stoichiometry revealed a significant shift in microbial metabolic strategies. The decrease in vector length (Figure 2A) indicated a reduction in microbial investment in carbon relative to other nutrients with increasing mulching duration. This finding aligns with the consistent decline in SOC (Table 1), suggesting that under long-term carbon limitation, microbial communities capable of synthesizing extracellular enzymes (BG, CBH) may have been progressively replaced by taxa employing alternative resource-use strategies. Meanwhile, the increase in VA (Figure 2B) clearly pointed to an enhancement of relative P limitation. This observation is particularly notable against the background of rising soil AP (Table 1), implying that microbial P acquisition constraints likely do not stem from an absolute lack of available P in the soil. Instead, they may be related to processes such as P speciation transformations, microbial biomass immobilization of P, or competitive phosphorus utilization within the soil-microbe-plant system. Furthermore, the sharp decline in N (TN, NH_4_^+^-N) content may have further intensified the relative microbial demand for P. VA of enzyme stoichiometry was greater than 45° (Figure 2A), indicating that microbial metabolism was predominantly constrained by P limitation. Although both TP and AP in soil showed an increasing trend with the duration of mulching (Table 1), the degree of P limitation continued to intensify. This seemingly contradictory phenomenon may be jointly driven by the following mechanisms: (1) adsorption of phosphate ions by MPs, which reduces the bioavailability of P; (2) enhanced microbial competition for P, particularly against the backdrop of increasing complexity in bacterial networks (Supplementary Table 2); and (3) inhibition of P transformation processes, such as the mineralization of organic P (Dong et al., 2020). Collectively, the increase in TP and AP in soil did not correspondingly improve microbial phosphorus use efficiency. Moreover, the presence of microplastics may further diminish the bioavailable P pool by altering the soil microenvironment or directly adsorbing phosphate (Liu et al., 2017).

The use of film coverings speeds up the breakdown of soil organic matter, decreases the resources available for microorganisms to obtain phosphorus, and hinders the mineralization and bioavailability of phosphorus, thereby worsening phosphorus scarcity. Particularly in the context of prolonged corn cropping alongside MP pollution, the imbalance in the phosphorus cycle in soil not only hampers microbial growth and reproduction but also impacts how effectively plants absorb and utilize phosphorus, ultimately jeopardizing the productivity and stability of the entire ecosystem.

## 5 Conclusion

This study reveals that long-term plastic film mulching significantly increases the abundance of MPs in cornfield soils, predominantly of film-type and small size (0–0.5 mm). It also drives changes in soil properties: elevating moisture and salinity (EC), reducing SOC and TN, while increasing TP and AP. The accumulation of MPs and the resulting environmental changes profoundly reshape the soil microbial community: bacterial diversity and network complexity increase, exemplified by an elevated abundance of *Actinobacteria*, which exhibits stronger interspecies collaboration. In contrast, the fungal network experiences intensified competition, as indicated by the increased abundance of Basidiomycota. Moreover, stoichiometric analysis of soil extracellular enzymes reveals a growing co-limitation of C and P in microbial metabolism, primarily driven by the depletion of SOC and alterations in phosphorus dynamics induced by MPs. Consequently, long-term plastic mulching exacerbates microbial nutrient limitations through MPs-mediated changes in soil physicochemical properties and microbial community restructuring, posing potential risks to soil health. To balance agricultural production with ecological sustainability, it is crucial to promote the use of biodegradable mulching films. To mitigate the accumulation of MPs and its associated risks documented in this study, future agronomic practices should consider exploring and adopting sustainable alternatives, such as biodegradable mulching films, which are designed to minimize residual plastic pollution.

## Data Availability

The datasets presented in this study can be found in online repositories. The names of the repository/repositories and accession number(s) can be found in the article/Supplementary material.

## References

[B1] An Q. Wen C. Yan C. (2024). Meta-analysis reveals the combined effects of microplastics and heavy metal on plants. *J. Hazard. Mater.* 476:135028. 10.1016/j.jhazmat.2024.135028 38925057

[B2] Bai Y. Gao J. (2021). Optimization of the nitrogen fertilizer schedule of maize under drip irrigation in Jilin, China, based on DSSAT and GA. *Agric. Water Manag.* 244:106555. 10.1016/j.agwat.2020.106555

[B3] Büks F. Kaupenjohann M. (2020). Global concentrations of microplastics in soils – a review. *SOIL* 6 649–662. 10.5194/soil-6-649-2020

[B4] Cao J. Gao X. Hu Q. Li C. Song X. Cai Y. (2023). Distribution characteristics and correlation of macro- and microplastics under long-term plastic mulching in northwest China. *Soil Tillage Res.* 231:105738. 10.1016/j.still.2023.105738

[B5] Cao Y. Shen Z. Zhang N. Deng X. Thomashow L. S. Lidbury I. (2024). Phosphorus availability influences disease-suppressive soil microbiome through plant-microbe interactions. *Microbiome* 12:185. 10.1186/s40168-024-01906-w 39342390 PMC11439275

[B6] Chang X. Fang Y. Wang Y. Wang F. Shang L. Zhong R. (2022). Microplastic pollution in soils, plants, and animals: A review of distributions, effects and potential mechanisms. *Sci. Total Environ.* 850:157857. 10.1016/j.scitotenv.2022.157857 35932864

[B7] Chen L. Yu L. Han B. Li Y. Zhang J. Tao S. (2024). Pollution characteristics and affecting factors of phthalate esters in agricultural soils in mainland China. *J. Hazard. Mater.* 466:133625. 10.1016/j.jhazmat.2024.133625 38295727

[B8] Clinton M. Rowe R. K. (2024). Long-term durability of two HDPE geomembranes formulated with polyethylene of raised temperature resistance (PE-RT). *Geotext. Geomembr.* 52 304–318. 10.1016/j.geotexmem.2023.11.003

[B9] Cui J. Zhang S. Wang X. Xu X. Ai C. Liang G. (2022). Enzymatic stoichiometry reveals phosphorus limitation-induced changes in the soil bacterial communities and element cycling: Evidence from a long-term field experiment. *Geoderma* 426:116124. 10.1016/j.geoderma.2022.116124

[B10] Cui Y. Zhang Y. Duan C. Wang X. Zhang X. Ju W. (2020). Ecoenzymatic stoichiometry reveals microbial phosphorus limitation decreases the nitrogen cycling potential of soils in semi-arid agricultural ecosystems. *Soil Tillage Res.* 197:104463. 10.1016/j.still.2019.104463

[B11] Dong Y. Gao M. Song Z. Qiu W. (2020). Microplastic particles increase arsenic toxicity to rice seedlings. *Environ. Pollut.* 259:113892. 10.1016/j.envpol.2019.113892 31931412

[B12] Fei Y. Huang S. Zhang H. Tong Y. Wen D. Xia X. (2020). Response of soil enzyme activities and bacterial communities to the accumulation of microplastics in an acid cropped soil. *Sci. Total Environ.* 707:135634. 10.1016/j.scitotenv.2019.135634 31761364

[B13] Gao L. Wang B. Li S. Wu H. Wu X. Liang G. (2019). Soil wet aggregate distribution and pore size distribution under different tillage systems after 16 years in the Loess Plateau of China. *CATENA* 173 38–47. 10.1016/j.catena.2018.09.043

[B14] Golmohammadi M. Fatemeh Musavi S. Habibi M. Maleki R. Golgoli M. Zargar M. (2023). Molecular mechanisms of microplastics degradation: A review. *Sep. Purif. Technol.* 309:122906. 10.1016/j.seppur.2022.122906

[B15] Huang Y. Liu Q. Jia W. Yan C. Wang J. (2020). Agricultural plastic mulching as a source of microplastics in the terrestrial environment. *Environ. Pollut.* 260:114096. 10.1016/j.envpol.2020.114096 32041035

[B16] Huang Y. Sheth R. U. Zhao S. Cohen L. A. Dabaghi K. Moody T. (2023). High-throughput microbial culturomics using automation and machine learning. *Nat. Biotechnol.* 41 1424–1433. 10.1038/s41587-023-01674-2 36805559 PMC10567565

[B17] Ji J. Peng L. Gao T. Salama E.-S. Khan A. Liu P. (2023). Microplastics enhanced the toxic effects of sulfamethoxazole on aerobic granular sludge and enriched antibiotic resistance genes. *Chem. Eng. J.* 464:142783. 10.1016/j.cej.2023.142783

[B18] Li J. Wu J. Yu J. Wang K. Li J. Cui Y. (2024). Soil enzyme activity and stoichiometry in response to precipitation changes in terrestrial ecosystems. *Soil Biol. Biochem.* 191:109321. 10.1016/j.soilbio.2024.109321

[B19] Li Y. Ma J. Li Y. Shen X. Xia X. (2024). Global change factors caused the decoupling of nutrient dynamics and asynchrony of microbial community and ecological function in temperate grassland soil. *Pedosphere* 35 627–640. 10.1016/j.pedsph.2024.05.007

[B20] Liu H. Yang X. Liu G. Liang C. Xue S. Chen H. (2017). Response of soil dissolved organic matter to microplastic addition in Chinese loess soil. *Chemosphere* 185 907–917. 10.1016/j.chemosphere.2017.07.064 28747000

[B21] Liu M. Lu S. Song Y. Lei L. Hu J. Lv W. (2018). Microplastic and mesoplastic pollution in farmland soils in suburbs of Shanghai, China. *Environ. Pollut.* 242 855–862. 10.1016/j.envpol.2018.07.051 30036839

[B22] Liu Y. Chen S. Zhou P. Li H. Wan Q. Lu Y. (2024). Differential impacts of microplastics on carbon and nitrogen cycling in plant-soil systems: A meta-analysis. *Sci. Total Environ.* 948:174655. 10.1016/j.scitotenv.2024.174655 39004375

[B23] Lu S. Hao J. Yang H. Chen M. Lian J. Chen Y. (2023). Earthworms mediate the influence of polyethylene (PE) and polylactic acid (PLA) microplastics on soil bacterial communities. *Sci. Total Environ.* 905:166959. 10.1016/j.scitotenv.2023.166959 37696400

[B24] Ma J. Cao Y. Fan L. Xie Y. Zhou X. Ren Q. (2023). Degradation characteristics of polybutylene adipate terephthalic acid (PBAT) and its effect on soil physicochemical properties: A comparative study with several polyethylene (PE) mulch films. *J. Hazard. Mater.* 456:131661. 10.1016/j.jhazmat.2023.131661 37224714

[B25] Ma J. Chen L. Pang D. Chen Y. Wu M. Zhang Y. (2025). Responses of soil microbial community structure under litter to changes in precipitation and nitrogen addition in a desert steppe. *Eur. J. Soil Biol.* 124:103696. 10.1016/j.ejsobi.2024.103696

[B26] Mondol M. Angon P. B. Roy A. (2024). Effects of microplastics on soil physical, chemical and biological properties. *Nat. Hazards Res.* 5 14–20. 10.1016/j.nhres.2024.02.002

[B27] Mujakić I. Piwosz K. Koblížek M. (2022). Phylum gemmatimonadota and its role in the environment. *Microorganisms* 10:151. 10.3390/microorganisms10010151 35056600 PMC8779627

[B28] Qi Y. Yang X. Pelaez A. M. Huerta Lwanga E. Beriot N. Gertsen H. (2018). Macro- and micro- plastics in soil-plant system: Effects of plastic mulch film residues on wheat (*Triticum aestivum*) growth. *Sci. Total Environ.* 645 1048–1056. 10.1016/j.scitotenv.2018.07.229 30248830

[B29] Qiu C. Zhou Y. Wang H. Chu Y. Zheng L. Chen Y. (2023). Microplastics enrichment characteristics of antibiotic resistance genes and pathogens in landfill leachate. *Chemosphere* 341:140100. 10.1016/j.chemosphere.2023.140100 37683946

[B30] Ren X. Tang J. Liu X. Liu Q. (2020). Effects of microplastics on greenhouse gas emissions and the microbial community in fertilized soil. *Environ. Pollut.* 256:113347. 10.1016/j.envpol.2019.113347 31672352

[B31] Sajjad M. Huang Q. Khan S. Khan M. A. Liu Y. Wang J. (2022). Microplastics in the soil environment: A critical review. *Environ. Technol. Innov.* 27:102408. 10.1016/j.eti.2022.102408

[B32] Shah T. Ali A. Haider G. Asad M. Munsif F. (2023). Microplastics alter soil enzyme activities and microbial community structure without negatively affecting plant growth in an agroecosystem. *Chemosphere* 322:138188. 10.1016/j.chemosphere.2023.138188 36804631

[B33] Song J. Chen X. Li S. Tang H. Dong S. Wang M. (2024). The environmental impact of mask-derived microplastics on soil ecosystems. *Sci. Total Environ.* 912:169182. 10.1016/j.scitotenv.2023.169182 38092201

[B34] Steinmetz Z. Wollmann C. Schaefer M. Buchmann C. David J. Tröger J. (2016). Plastic mulching in agriculture. Trading short-term agronomic benefits for long-term soil degradation? *Sci. Total Environ.* 550 690–705. 10.1016/j.scitotenv.2016.01.153 26849333

[B35] Sun Q. Zhang X. Liu C. Ying S. Zhang J. (2023). The content of PAEs in field soils caused by the residual film has a periodical peak. *Sci. Total Environ.* 864:161078. 10.1016/j.scitotenv.2022.161078 36565862

[B36] Surendran U. Jayakumar M. Raja P. Gopinath G. Chellam P. V. (2023). Microplastics in terrestrial ecosystem: Sources and migration in soil environment. *Chemosphere* 318:137946. 10.1016/j.chemosphere.2023.137946 36708782

[B37] Tang Y. Xing Y. Wang X. Ya H. Zhang T. Lv M. (2024). PET microplastics influenced microbial community and heavy metal speciation in heavy-metal contaminated soils. *Appl. Soil Ecol.* 201:105488. 10.1016/j.apsoil.2024.105488

[B38] Thompson R. C. Olsen Y. Mitchell R. P. Davis A. Rowland S. J. John A. W. G. (2004). Lost at sea: Where is all the plastic? *Science* 304:838. 10.1126/science.1094559 15131299

[B39] Wang J. Wu Y. Li J. He Q. Bing H. (2021). Soil enzyme stoichiometry is tightly linked to microbial community composition in successional ecosystems after glacier retreat. *Soil Biol. Biochem.* 162:108429. 10.1016/j.soilbio.2021.108429

[B40] Wang S. Fan T. Cheng W. Wang L. Zhao G. Li S. (2022). Occurrence of macroplastic debris in the long-term plastic film-mulched agricultural soil: A case study of Northwest China. *Sci. Total Environ.* 831:154881. 10.1016/j.scitotenv.2022.154881 35364156

[B41] Wang W. Zhang J. Qiu Z. Cui Z. Li N. Li X. (2022). Effects of polyethylene microplastics on cell membranes: A combined study of experiments and molecular dynamics simulations. *J. Hazard. Mater.* 429:128323. 10.1016/j.jhazmat.2022.128323 35086040

[B42] Xu L. Wang Y. Wei F. Dai Z. Zhang M. (2024). Transport behavior of microplastics in soil–water environments and its dependence on soil components. *Environ. Pollut.* 346:123542. 10.1016/j.envpol.2024.123542 38355087

[B43] Xue Y. Zhao F. Sun Z. Bai W. Zhang Y. Zhang Z. (2023). Long-term mulching of biodegradable plastic film decreased fungal necromass C with potential consequences for soil C storage. *Chemosphere* 337:139280. 10.1016/j.chemosphere.2023.139280 37385482

[B44] Yang Y. Tong Y. Liang L. Li H. Han W. (2021). Dynamics of soil bacteria and fungi communities of dry land for 8 years with soil conservation management. *J. Environ. Manage.* 299:113544. 10.1016/j.jenvman.2021.113544 34467869

[B45] Yu H. Fan P. Hou J. Dang Q. Cui D. Xi B. (2020). Inhibitory effect of microplastics on soil extracellular enzymatic activities by changing soil properties and direct adsorption: An investigation at the aggregate-fraction level. *Environ. Pollut.* 267:115544. 10.1016/j.envpol.2020.115544 32911337

[B46] Yu Y. Chen H. Chen G. Su W. Hua M. Wang L. (2024). Deciphering the crop-soil-enzyme C:N:P stoichiometry nexus: A 5-year study on manure-induced changes in soil phosphorus transformation and release risk. *Sci. Total Environ.* 934:173226. 10.1016/j.scitotenv.2024.173226 38768729

[B47] Yu Y. Zhang Z. Zhang Y. Jia H. Li Y. Yao H. (2023). Abundances of agricultural microplastics and their contribution to the soil organic carbon pool in plastic film mulching fields of Xinjiang, China. *Chemosphere* 316:137837. 10.1016/j.chemosphere.2023.137837 36640972

[B48] Zhang H. Huang Y. An S. Wang P. Xie C. Jia P. (2024). Mulch-derived microplastic aging promotes phthalate esters and alters organic carbon fraction content in grassland and farmland soils. *J. Hazard. Mater.* 461:132619. 10.1016/j.jhazmat.2023.132619 37757559

[B49] Zhang H. Huang Y. An S. Zhao J. Xiao L. Li H. (2022). Microplastics trapped in soil aggregates of different land-use types: a case study of Loess Plateau terraces, China. *Environ. Pollut.* 310:119880. 10.1016/j.envpol.2022.119880 35932900

[B50] Zhang M. Zhao G. Li Y. Wang Q. Dang P. Qin X. (2021). Straw incorporation with ridge–furrow plastic film mulch alters soil fungal community and increases maize yield in a semiarid region of China. *Appl. Soil Ecol.* 167:104038. 10.1016/j.apsoil.2021.104038

[B51] Zhang S. Wang J. Hao X. (2020). Fertilization accelerates the decomposition of microplastics in mollisols. *Sci. Total Environ.* 722:137950. 10.1016/j.scitotenv.2020.137950 32208279

[B52] Zhang Y. Li X. Xiao M. Feng Z. Yu Y. Yao H. (2022). Effects of microplastics on soil carbon dioxide emissions and the microbial functional genes involved in organic carbon decomposition in agricultural soil. *Sci. Total Environ.* 806:150714. 10.1016/j.scitotenv.2021.150714 34606872

[B53] Zhao S. Zhang Z. Chen L. Cui Q. Cui Y. Song D. (2022). Review on migration, transformation and ecological impacts of microplastics in soil. *Appl. Soil Ecol.* 176:104486. 10.1016/j.apsoil.2022.104486

[B54] Zhao Y. Xu Z. Mao X. Li S. Qi X. Che J. (2024). Influence of film color, mulching ratio and soil–mulch contact degree on heat transfer in Northwest China. *Agric. For. Meteorol.* 357:110208. 10.1016/j.agrformet.2024.110208

[B55] Zhou B. Wang J. Zhang H. Shi H. Fei Y. Huang S. (2020). Microplastics in agricultural soils on the coastal plain of Hangzhou Bay, east China: multiple sources other than plastic mulching film. *J. Hazard. Mater.* 388:121814. 10.1016/j.jhazmat.2019.121814 31843412

[B56] Zhou J. Bruns M. A. Tiedje J. M. (1996). DNA recovery from soils of diverse composition. *Appl. Environ. Microbiol.* 62 316–322. 10.1128/aem.62.2.316-322.1996 8593035 PMC167800

[B57] Zhou J. Xu H. Xiang Y. Wu J. (2024). Effects of microplastics pollution on plant and soil phosphorus: a meta-analysis. *J. Hazard. Mater.* 461:132705. 10.1016/j.jhazmat.2023.132705 37813034

[B58] Zhou X. Xiao C. Zhang B. Chen T. Yang X. (2024). Effects of microplastics on carbon release and microbial community in mangrove soil systems. *J. Hazard. Mater.* 465:133152. 10.1016/j.jhazmat.2023.133152 38056259

