# Supplementary material for: Long-term plastic mulching exacerbates the co-limitation of carbon and phosphorus in farmland by altering physicochemical properties and microbial interactions
Source: Front Microbiol. 2026 Jan 14;16:1694370. doi: 10.3389/fmicb.2025.1694370 (PMC12847442; doi:10.3389/fmicb.2025.1694370)
Supplement: Supplementary file 1 [file Table_1.docx]

***Supplementary material***

**Supplementary Figures and Tables**

**Supplementary Figures**


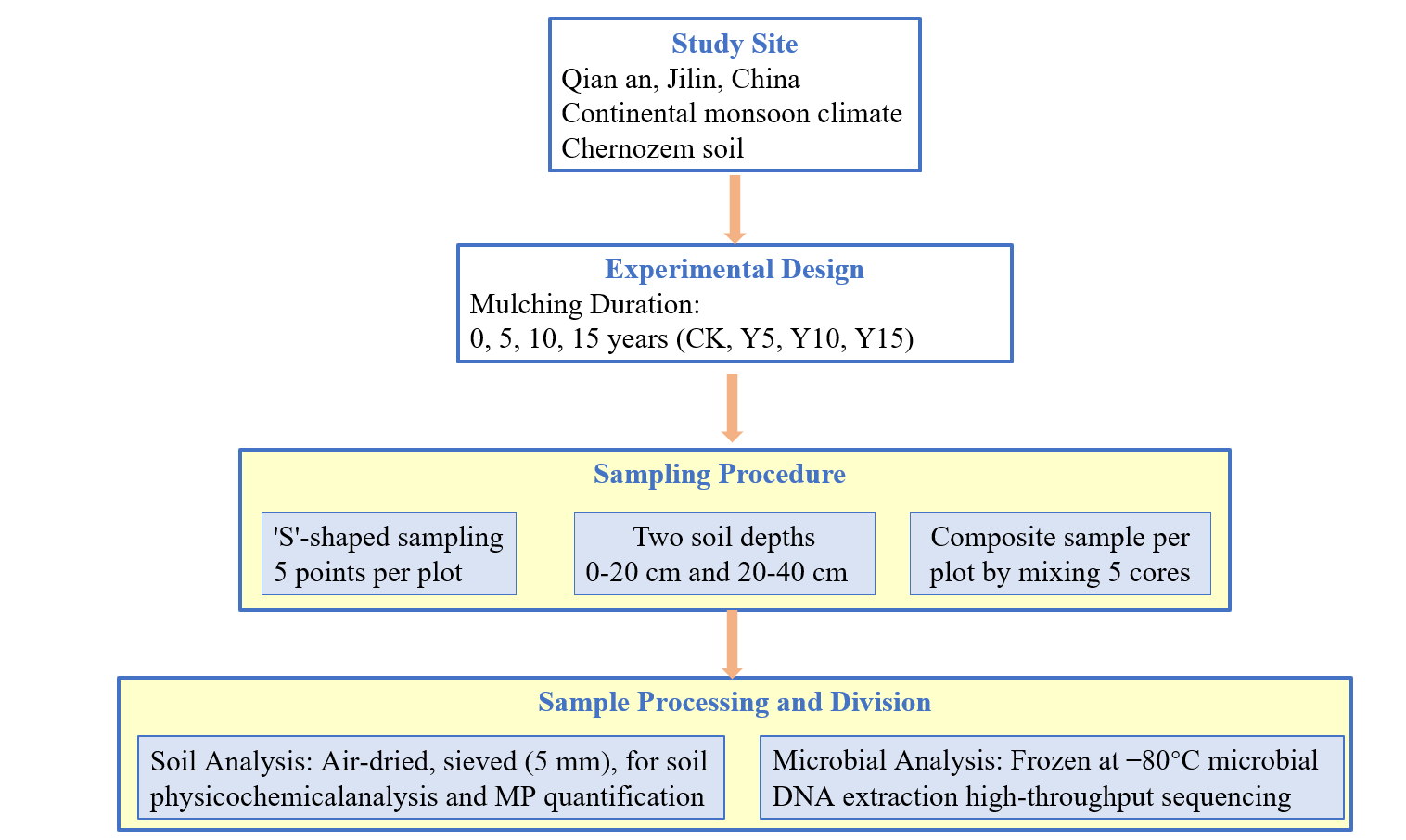


Fig. S1. Experimental flowchart.


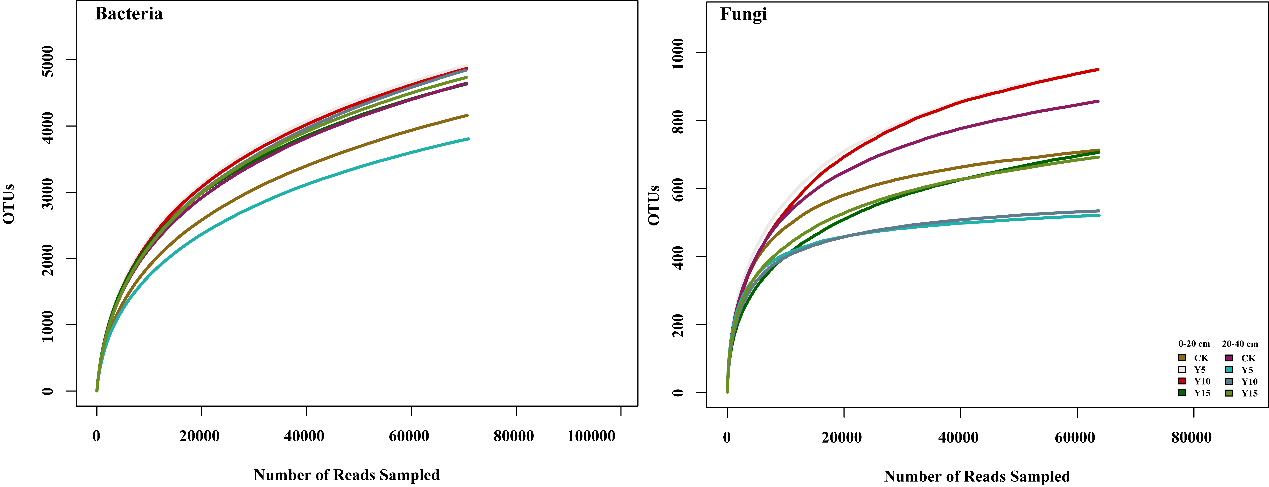


Fig. S2. Rarefaction curves under different mulching years.


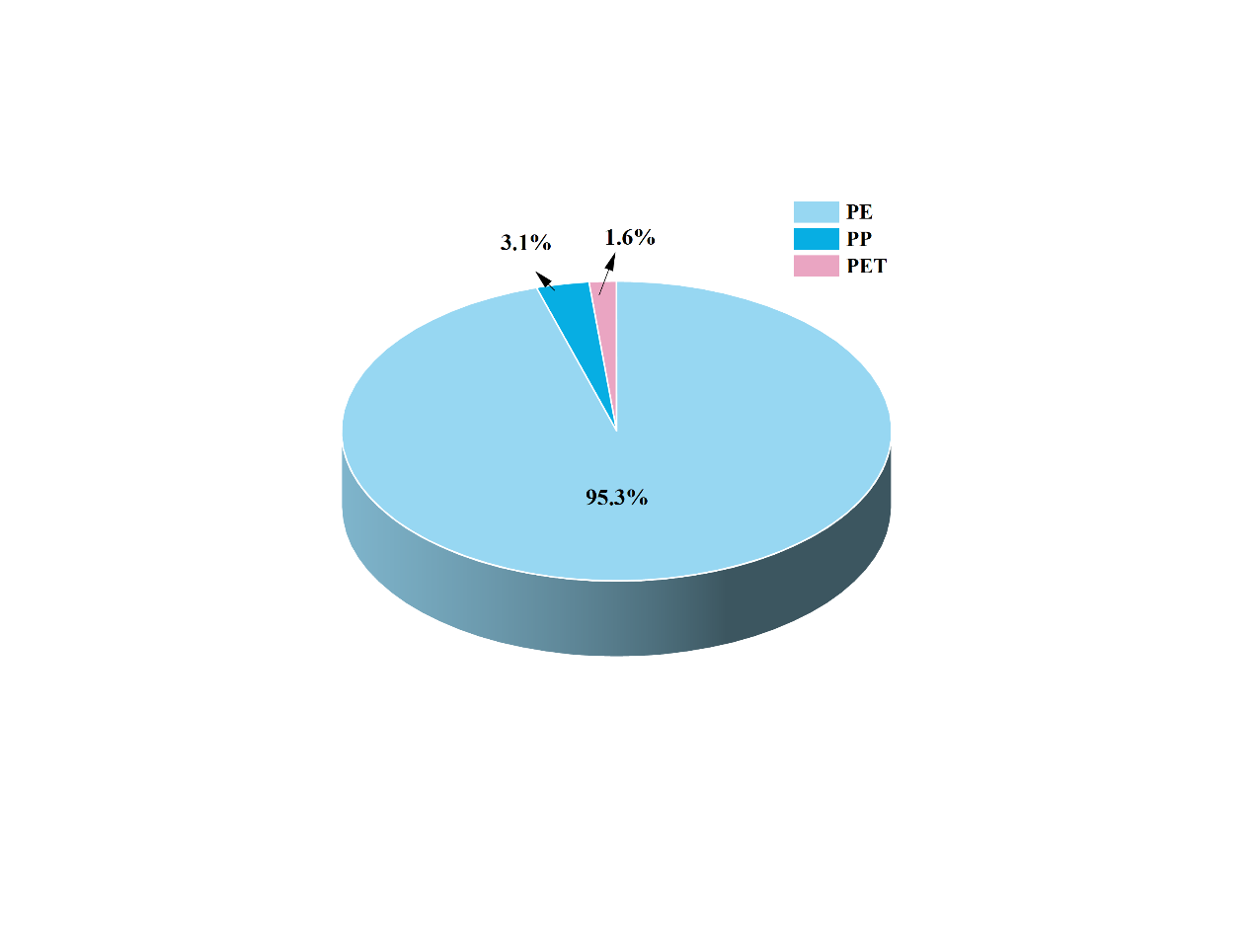


Fig. S3. Polymer composition of microplastics in soils of different treatments.


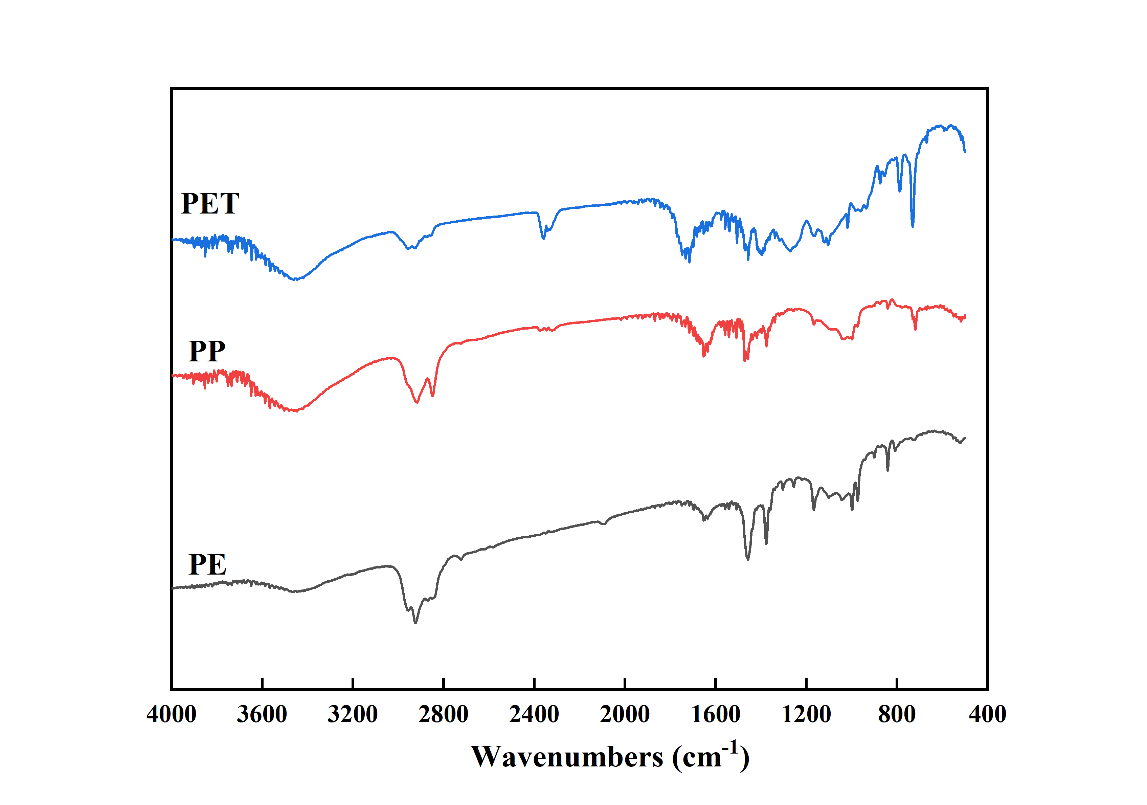


Fig. S4. FTIR spectra of microplastics from long-term plastic mulching experiment.

**
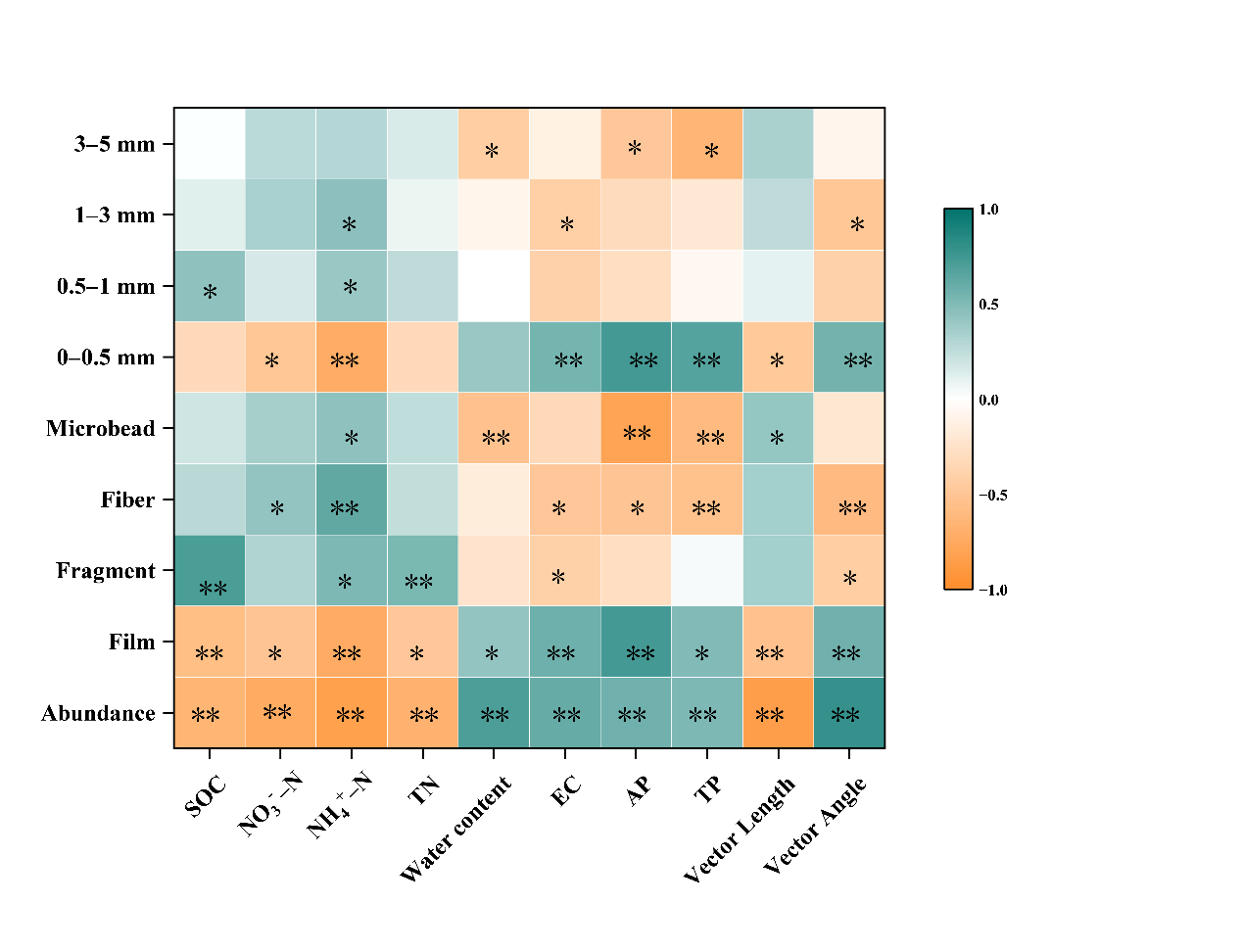
**

Fig. S5. Heatmap of correlation among soil MPs pollution (MPs shapes, sizes, abundance), microbial nutrient limitations and soil properties. * Correlation is significant at the 0.05 level; ** Correlation is significant at the 0.01 level.

**
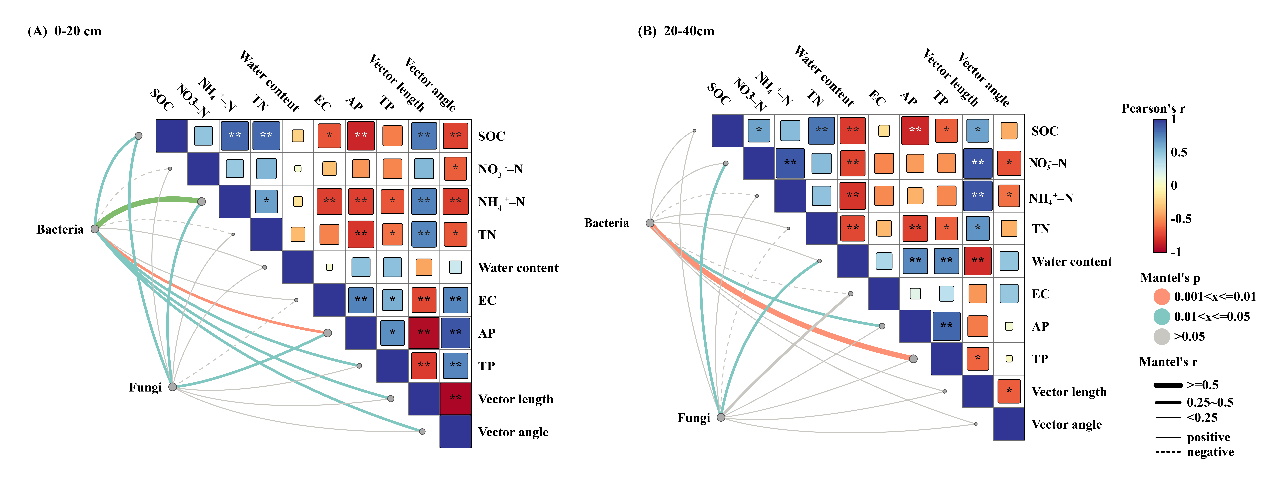
**

Fig. S6. Mantel test revealing the relationships between microbial community and properties of soil. * represents signiffcantly different at the 0.05 level; ** represents signiffcantly different at 0.01 level; *** represents signiffcantly different at 0.001 level. SOC: soil organic C; TN: total nitrogen; TP: total phosphorus; EC: electrical conductivity; AP: available phosphorous.

**Supplementary Tables**

Table S1 Description of Experimental Treatments

| Treatment | Planting Duration | Plastic Film Mulching Duration | Mulching Period | Management Description |
| --- | --- | --- | --- | --- |
| CK | 15 years | 0 years | - | Continuous corn planting, never mulched with plastic film |
| Y5 | 15 years | 5 years | 2019-2023 | Continuous corn planting, mulched in the recent 5 years |
| Y10 | 15 years | 10 years | 2014-2023 | Continuous corn planting, mulched in the recent 10 years |
| Y15 | 15 years | 15 years | 2008-2023 | Continuous corn planting, mulched in the recent 15 years |

Table S2 Topological indices of soil microbial networks under different mulching years (Bacteria).

| Soil Layer | Treatment | Node | Edge | Positive (P) (%) | Negative (N) (%) | P/N | Average clustering  coefficient | Average  degree | Average  path length | Network  diameter | Network  density | Modularity |
| --- | --- | --- | --- | --- | --- | --- | --- | --- | --- | --- | --- | --- |
| 0-20 cm | CK | 172 | 2497 | 50.51 | 49.49 | 1.20 | 0.63 | 29.035 | 2.613 | 7 | 0.17 | 0.370 |
|  | Y5 | 172 | 5239 | 53.07 | 46.93 | 1.13 | 0.73 | 61.547 | 2.654 | 7 | 0.36 | 0.379 |
|  | Y10 | 172 | 2612 | 53.38 | 46.62 | 1.15 | 0.69 | 29.395 | 2.692 | 7 | 0.177 | 0.448 |
|  | Y15 | 173 | 2512 | 74.43 | 25.57 | 2.91 | 0.64 | 29.075 | 2.649 | 8 | 0.179 | 0.386 |
| 20-40 cm | CK | 172 | 2055 | 58.78 | 41.22 | 1.43 | 0.60 | 23.893 | 2.739 | 6 | 0.14 | 0.413 |
|  | Y5 | 173 | 2971 | 53.93 | 46.07 | 1.17 | 0.62 | 22.786 | 2.803 | 8 | 0.132 | 0.451 |
|  | Y10 | 170 | 3784 | 51.77 | 48.23 | 1.07 | 0.70 | 44.518 | 2.352 | 8 | 0.263 | 0.176 |
|  | Y15 | 172 | 4051 | 58.55 | 41.45 | 1.41 | 0.71 | 47.105 | 2.48 | 7 | 0.275 | 0.259 |

Table S3 Topological indices of soil microbial networks under different mulching years (Fungi).

| Soil Layer | Treatment | Node | Edge | Positive (%) | Negative (%) | P/N | Average clustering  coefficient | Average degree | Average  path length | Network diameter | Network density | Modularity |
| --- | --- | --- | --- | --- | --- | --- | --- | --- | --- | --- | --- | --- |
| 0-20 cm | CK | 127 | 602 | 82.89 | 17.11 | 4.84 | 0.56 | 9.48 | 3.263 | 8 | 0.075 | 0.519 |
|  | Y5 | 129 | 851 | 62.16 | 37.84 | 1.64 | 0.64 | 13.194 | 3.175 | 9 | 0.103 | 0.485 |
|  | Y10 | 126 | 571 | 59.89 | 40.11 | 1.49 | 0.59 | 9.063 | 3.352 | 7 | 0.073 | 0.622 |
|  | Y15 | 126 | 495 | 54.66 | 45.34 | 1.20 | 0.48 | 7.841 | 3.151 | 6 | 0.063 | 0.563 |
| 20-40 cm | CK | 126 | 822 | 83.58 | 16.42 | 5.10 | 0.57 | 13.048 | 2.937 | 7 | 0.104 | 0.509 |
|  | Y5 | 127 | 493 | 60.24 | 39.76 | 1.52 | 0.54 | 7.764 | 3.354 | 6 | 0.062 | 0.626 |
|  | Y10 | 129 | 572 | 62.59 | 37.41 | 1.67 | 0.52 | 8.868 | 3.148 | 6 | 0.069 | 0.6 |
|  | Y15 | 123 | 563 | 53.46 | 46.54 | 1.149 | 0.56 | 9.154 | 3.127 | 6 | 0.075 | 0.59 |
